# Identification and Characterization of a Novel Phosphodiesterase from the Metagenome of an Indian Coalbed

**DOI:** 10.1371/journal.pone.0118075

**Published:** 2015-02-06

**Authors:** Durgesh Narain Singh, Ankush Gupta, Vijay Shankar Singh, Rajeev Mishra, Suneel Kateriya, Anil Kumar Tripathi

**Affiliations:** 1 School of Biotechnology, Faculty of Science, Banaras Hindu University, Varanasi—221005, Uttar Pradesh, India; 2 Bioinformatics programme, Mahila Maha Vidyalaya, Banaras Hindu University, Varanasi—221005, Uttar Pradesh, India; 3 Department of Biochemistry, University of Delhi South Campus, Benito Juarez Road, New Delhi-110021, India; New England Biolabs, Inc., UNITED STATES

## Abstract

Phosphoesterases are involved in the degradation of organophosphorus compounds. Although phosphomonoesterases and phosphotriesterases have been studied in detail, studies on phosphodiesterases are rather limited. In our search to find novel phosphodiesterases using metagenomic approach, we cloned a gene encoding a putative phosphodiesterase (PdeM) from the metagenome of the formation water collected from an Indian coal bed. Bioinformatic analysis showed that PdeM sequence possessed the characteristic signature motifs of the class III phosphodiesterases and phylogenetic study of PdeM enabled us to identify three distinct subclasses (A, B, and C) within class III phosphodiesterases, PdeM clustering in new subclass IIIB. Bioinformatic, biochemical and biophysical characterization of PdeM further revealed some of the characteristic features of the phosphodiesterases belonging to newly described subclass IIIB. PdeM is a monomer of 29.3 kDa, which exhibits optimum activity at 25°C and pH 8.5, but low affinity for bis(*p*NPP) as well as *p*NPPP. The recombinant PdeM possessed phosphodiesterase, phosphonate-ester hydrolase and nuclease activity. It lacked phosphomonoesterase, phosphotriesterase, and RNAse activities. Overexpression of PdeM in *E.coli* neither affected catabolite respression nor did the recombinant protein hydrolyzed cAMP *in vitro*, indicating its inability to hydrolyze cAMP. Although Mn^2+^ was required for the activity of PdeM, but addition of metals (Mn^2+^ or Fe^3+^) did not induce oligomerization. Further increase in concentration of Mn^2+^ upto 3 mM, increased α-helical content as well as the phosphodiesterase activity. Structural comparison of PdeM with its homologs showed that it lacked critical residues required for dimerization, cAMP hydrolysis, and for the high affinity binding of bis(*p*NPP). PdeM, thus, is a novel representative of new subclass of class III phosphodiesterases.

## Introduction

Phosphorus is an essential element which is present as organophosphates in a variety of biological molecules such as DNA, RNA, phospholipids, phosphorylated proteins, cyclic nulceotides monophosphates etc in the cell [1, 2]. Bacterial catabolic pathway for the degradation of a typical organophosphate (phosphotriester) compound to an inorganic phosphate involves three hydrolytic enzymes. Phosphotriesterases hydrolyze phosphotriester compounds into phosphodiester compounds, phosphodiesterases hydrolyze phosphodiester compounds into phosphomonoesters, and eventually phosphomonoesterases hydrolyze phosphomonoesters into phosphates [3]. Phosphomonoesterases including alkaline and acid phosphatases have been investigated in detail [4, 5]. Phosphotriesterases have also been investigated extensively due to their potential in detoxifying organophosphate pesticides and nerve agents [6]. Phosphodiesters, including nucleic acids, are chemically more resistant to hydrolysis than phosphotriesters [7]. But, due to the non-toxic nature of phosphodiesters, phosphodiesterases are the least studied of all phosphoesterases [7].

The phosphoesterases catalyzing the hydrolysis of phosphate monoesters, diesters and triesters are known as metallophosphoesterases (MPEs) because their active site coordinates two metal ions: Fe^3+^, Mn^2+^, Ni^2+^ or Zn^2+^ at site 1, and Fe^2+^, Zn^2+^ or Mn^2+^ at site 2 with octahedral geometry using two aspartic acid, one asparagine and four histidine residues [8,9,10]. MPEs are structurally related proteins, which constitute a super family consisting of calcineurin-like phosphoesterases with diverse functions, and are believed to have evolved from a common ancestor [11]. Structural similarity between the active sites of mono- and diesterases suggests that bimetallo active sites might be able to catalyze either type of reactions [12]. The esterifying group in a diester causes steric clash within the active site of a monoesterase reducing its affinity towards diesters [13]. The catalytic machinery required for the hydrolysis of phosphodiesters and phosphotriesters is also very similar, as an alteration of only one amino acid can confer phosphodiesterase activity to a phosphotriestrase [7].

There are many families of phosphodiesterases, which include phospholipases C and D, autotaxin, sphingomyelin phosphodiesterase, DNases, RNases, and restriction endonucleases. However, phosphodiesterases (PDEs) usually refer to the cyclic nucleotide phosphodiesterases, which comprise a group of enzymes that degrade phosphodiester bond in cAMP and cGMP [9]. Based on their primary structure and differences in their catalytic domain, PDEs were previously divided into the two main classes, Class I and Class II, which were characterized by H(X)_3_(X)_25–35_D/E and HXHLDH motifs, respectively [9]. Class I PDEs are found mainly in higher eukaryotes whereas Class II PDEs have been found in organisms such as yeast, *Dictyostelium* and *Vibrio* [9]. Since a 3’, 5’-cyclic AMP phosphodiesterase from *E*.*coli* (encoded by *cpdA* gene) [14] lacked any homology to Class I or Class II PDEs, it represented a new class of PDEs, class III PDE, which showed catalytic features of the purple acid phosphatases and dimetallophosphoesterases having a conserved sequence motif, D(X)H(X)_n_GD(X)_n_GNHD(X)_n_H(X)nGH(X)H [9, 10]. Another phosphodiesterase, PdeA was later cloned and characterized from *Delftia acidovoran*s, which grew on phosphodiester compounds as sole source of phosphorus [1]. Rv0805, a PDE from *M*. *tuberculosis* is the only Class III PDE, which has been characterized in greater detail [10, 15]. Both, CpdA and PdeA were structurally similar to Rv0805 as evident from their common sequence signature D(X)H-(X)_37–38_-GD-(X)_28–32_-GNH[E/D]-(X)_68–70_-H-(X)_35–37_-GH(X)H.

Probability of finding novel genes and enzymes from organisms residing an extreme habitat such as coal bed is undoubtedly high. Coal is derived from fossilized plants that are transformed into hydrocarbons *via* thermogenic or biogenic activities in the coalbed over a long period of time [16]. Whereas simple carbon compounds are transformed into fossil fuels including methane, relatively more complex compounds tend to accumulate in the coalbeds affecting cycling of essential nutrients including phosphorus [17]. During the process of production of coalbed methane (a natural gas), large volumes of water (known as formation water) are pumped out of the coalbed [17, 18]. In our search for novel genes and enzymes, we have identified, cloned and characterized a novel phosphodiesterase from the metagenome of the formation water collected from an Indian coalbed. In this study phylogenetic analysis of the metagenomic phosphodiesterase (PdeM) revealed that class III PDEs consist of three distinct subclasses. Bioinformatic, biochemical and biophysical characterization of PdeM elucidates, for the first time, some of the unique structural and functional features of a new subclass of class III PDEs.

## Materials and Methods

### Bacterial strains, culture conditions, plasmids and chemicals

*Escherichia coli* DH5α (Gibco/BRL) and *E*. *coli* BL21 (DE3) (Novagen) were grown in Luria-Bertani (LB) medium at 37°C in the presence of appropriate antibiotics where required. *E*. *coli* DH5α was used as a cloning host and *E*. *coli* BL21 (DE3) was used as expression host. Fosmid pCC2FOS (Epicenter) and plasmid pET15b (Novagen) were used as vectors. All chemicals used for growing bacteria were from Hi-media (Mumbai, India), substrates used for enzymatic assays were purchased from Sigma Chemical Co, and enzymes used for DNA modification and cloning were from New England Biolab.

### Construction of metagenomic DNA library and identification of clones hydrolyzing bis(*p*NPP)

The metagenomic DNA was extracted from the formation water of a coalbed located in Jharia coalfield (Jharia, Jharkhand, India; 23.75°N 86.42°E) [19]. For cloning the large fragments of metagenomic DNA, a fosmid library was constructed in pCC2FOS fosmid vector from the metagenomic DNA as per the protocol of the Copy-Control Fosmid Library Production Kit (Epicenter). Briefly, purified metagenomic DNA was fragmented by passing through a 200 μl small-bore pipette tip for 3 times. Gel electrophoresis was performed at 35 V for 12 h, and then approximately 35–40 kb DNA fragment were isolated from the gel. DNA was end-repaired in reaction containing 40 μl DNA (0.5 μg μl^-1^), 8 μl 10X end-repair buffer, 8 μl 2.5 mM dNTP mix, 8 μl 10 mM ATP, 4 μl end-repair enzyme mix (Epicenter) and 12 μl sterile deionized water. Reaction mixture was incubated at room temperature for 1 h. Gel loading buffer was added to this reaction mix, and end-repair enzyme was inactivated by heating at 70°C for 10 min. The end-repaired DNA was loaded in low-melting agarose gel, and DNA of approximately 40 kb was eluted. This end-repaired, blunt-ended and 5’phosphorylated DNA was then ligated to a copycontrol pCC2FOS^TM^ vector. Lambda packaging extracts were then added to this ligation mix, and used for the infection of a T1 phage-resistant *E*. *coli* strain (EPI300-T1). Transformants were selected on chloramphenicol (12.5 μg mL^-1^) amended LB agar plates. Selected transformants (208) were then replica plated on LB agar plates supplemented with bis(*p*NPP) and incubated at 37°C overnight. One of the transformants showed a yellow colored halo indicating hydrolysis of bis(*p*NPP), and suggesting the presence of a phosphodiesterase gene.

### Sequencing and bioinformatic analysis

For the isolation of fosmid DNA showing hydrolysis of bis(*p*NPP) the selected clone was grown overnight in LB broth containing chloramphenicol (12.5 μg mL^-1^) and autoinduction (1μl mL^-1^) solution (Epicenter). The fosmid DNA was isolated using fosmid DNA isolation kit (Epicentre), digested with *EcoR*1 and resolved in agarose gel to confirm the presence of insert in the fosmid vector. The selected fosmid DNA was sequenced (Chromous biotech), and analyzed for genes/ ORFs using fgenesh B software (http://linux1.softberry.com/berry.phtml) of gene prediction, and the nucleotide sequence was submitted to NCBI GenBank (GI: 528880712; Accession number: KF478243).

For phylogenetic analysis, PDE sequences were retrieved from the NCBI using PSI Blast tool against the Refseq database [20]. Non-redundant set of sequences selected from diverse organisms is shown in S1 Table. A multiple sequence alignment was generated using ClustalX [21], and the neighbor-joining (NJ) tree constructed using MEGA software version 5.0. The search for suitable templates to model PdeM was carried out using HHpred tool [22]. Secondary structure of the metagenomic phosphodiesterase (designated as PdeM) was predicted with PSIPRED tool [23]. The homology model of PdeM, based on the three chosen templates, was constructed by using the multi-template modeling module of Modeller 9v2 tool [24], which utilizes an automated approach for comparative protein structure modeling by the satisfaction of spatial restraints. The Ramachandran plot was produced with PROCHECK [25].

### Cloning, overexpression and purification of metagenomic phosphodiesterase

A construct for heterologous expression of the gene encoding metagenomic phosphodiesterase (PdeM) was constructed by PCR amplification from the fosmid clone and cloning into the expression vector pET15b (Novagen). The complete coding region of the PdeM gene was amplified by PCR using primers pdemF/pdemR designed from the annotated gene sequence (Froward 5’-GGGAATTCCATATGAGCCCCCAAGCTTCACCG-3’ and Reverse 5’-CGCGGATCCTCAAGTTCCCGGCAAGGCCC-3’). These primers introduce restriction sites for NdeI/BamHI (underline). PCR amplification reactions were performed with a Veriti 96-well Thermal cycler (Applied Biosystem, USA) using the following cycles: an initial denaturation at 95°C for 5 min; 35 cycles of denaturation at 94°C for 1 min, annealing at 57°C for 1 min and extension at 72°C for 1 min with single extension cycle at 72°C for 5 min before cooling at 4°C. The amplicon of 747 bp was digested with NdeI/BamHI restriction enzymes, purified with PCR purification kit (Promega) and finally ligated with the similarly digested expression vector pET15b (Novagen) to generate the recombinant plasmid, pET-PdeM. *E*. *coli* DH5α was then transformed with the ligation mix and the transformants were selected on Luria agar with ampicillin (100 μg mL^-1^). After confirmation of the clones by restriction digestion and sequencing, *E*. *coli* BL21 (DE3) competent cells were transformed with the recombinant plasmid and the transformants were selected on Luria agar amended with ampicillin (100 μg mL^-1^).

For overexpression and purification of recombinant protein, the recombinant *E*. *coli* BL21 (DE3) cells grown overnight in LB medium containing ampicillin (100 μg mL^-1^) at 37°C were inoculated in fresh LB medium (1:100) containing ampicillin (100 μg mL^-1^) and incubated at 37°C with shaking at 170 rpm. When A_600_ nm reached a value of ~0.6–0.8, the expression of His-tagged metagenomic protein was induced by adding 0.5 mM isopropyl β-D-thiogalactoside, cells were grown for 20 h at 16°C under shaking condition of 170 rpm. The cells were harvested by centrifugation and resuspended in lysis buffer (50 mM Tris-Cl, pH.8.0, 300 mM NaCl, 10 mM imidazole, 1mM phenylmethylsulfonyl fluoride, 0.1% Triton X-100), lysed with lysozyme (1 mg mL^-1^) on ice for 1h, followed by sonication at 4°C with six bursts of 9 s each and a cooling period of 11 s between each burst. The lysate was centrifuged (10 000 g for 10 min at 4°C), the resultant supernatant was added to pre-equilibrated Ni-NTA resin (Qiagen) and mixed gently with shaking for 1 h. The lysate-Ni-NTA mixture was loaded on the column and the non- specifically bound proteins were removed by washing twice with wash buffer (50 mM Tris-Cl, pH 8.0, 300 mM NaCl, 50 mM imidazole). The bound fusion protein was eluted with elution buffer (50 mM Tris-Cl, pH 8.0, 300 mM NaCl, and 250 mM imidazole). All purification steps were carried out at 4°C. The homogeneity of the purified protein was checked by loading on 12% SDS-PAGE and staining by Coomassie Brilliant Blue R-250. Purified protein was stored at -80°C until further use.

### Size-exclusion chromatography

For determination of the oligomeric status, the affinity purified protein was extracted from *E*. *coli* BL21 (DE3) harboring pET-PdeM plasmid grown in LB medium with or without 100 μM each of Mn^2+^, Fe ^3+^ or both. Sterile stock solutions of metal ions were prepared and added to the autoclaved LB medium prior to inoculation. The oligomeric state of the affinity purified protein was confirmed by loading ~2.0 mg mL^-1^ of the purified proteins on Superdex 200 10/300 GL gel permeation column (GE Healthcare) in a buffer (Tris.Cl (pH 7.5) 25 mM, NaCl 50 mM, β-ME 5 mM) containing 10 μM of the respective metal ions. Fractions of 1.0 mL were collected at a flow rate of 0.5 mL min^-1^ and assayed for total protein concentration and activity [26]. Four protein standards were used (thyroglobulin, Mr 669 kDa, aldolase, Mr 158 kDa, conalbumin, Mr 75 kDa and albumin, Mr 44 kDa) (Gel filtration HMW calibration Kit, GE Healthcare) to prepare molecular weight calibration curve.

### Screening of substrates for enzymatic activities of PdeM

The PdeM protein was screened for its catalytic activities such as phosphatase, phosphodiesterase and nuclease [27]. Phosphatase activity was estimated using p-nitrophenyl phosphate (40 mM) as substrate in 1 mL reaction volume containing 50 mM Tris.Cl (pH 8.5) or HEPES-K (pH 7.5), 5 mM MgCl_2_, and 1 mM MnCl_2_. The reaction was initiated by adding 1 μg PdeM protein incubated at 25°C for 10 min, followed by measuring absorbance at 405 nm [27]. Phosphodiesterase activity was analyzed using 4 mM each of bis *p*-nitrophenyl phosphate [bis(*p*NPP)], *p-*nitrophenyl phenylphosphonate (*p*NPPP), o-(4-nitrophenyl phosphoryl) choline (*p*NPPC) and thymidine 5’-monophosphate p-nitrophenyl ester (*p*NP-TMP) (all Sigma-Aldrich) as chromogenic substrates in a reaction volume of 1 mL each containing 50 mM Tris.Cl (pH 8.5), 5 mM β-ME and 10 mM NaCl in the absence or presence of 2mM Mn^2+^ ions.

Phosphodiesterase activity of PdeM was also screened for cNMP’s *viz*; 2’3’cAMP, 3’5’cAMP, 3’5’cGMP or c-di-GMP (all Sigma-Aldrich) [28, 29]. Briefly, 1 mL of reaction mixture containing 50 mM Tris.Cl (pH 8.0), 5–20 μM of cNMP substrates and 5 μg PdeM was incubated in the absence or presence of 2 mM Mn^2+^ ions at 37°C for 1 hour. The reaction was stopped by boiling the sample in water bath for 2 min, and the protein precipitate was removed by centrifugation. Supernatant of the reactions were analyzed by LC-MS/MS to separate cNMPs from 5’NMPs [28]. All the experiments were performed at least three times with three different lots of protein prepared independently on separate days. Phosphotriesterase activity was carried out in 1 mL reaction volume in 50 mM Tris.Cl buffer (pH 8.5) as well as Tris-glycine buffer (pH 8.5) containing 5 mM MgCl_2_, 1 mM MnCl_2_ and 5 mM methyl parathion. The reaction was initiated by adding 1 μg protein and incubated at 25°C for 1 hour followed by measuring absorbance at 405 nm [30].

Nuclease activity of PdeM was screened on single-stranded DNA (26 mer synthetic oligo), double stranded linear DNA, plasmid DNA (pET21a), genomic DNA from *E*. *coli* and total ribosomal RNA (*A*. *brasilense* Sp7), and the reaction products were analyzed by agarose gel- and polyacrylamide gel electrophoresis [27]. A 20 μl reaction mixture containing 50 mM Tris.Cl (pH 8.0), 4 μg of the above substrates and 5 μg of PdeM protein was incubated at 37°C for 1 hour, in the absence or presence of 2 mM Mn^2+^ ions.

### Kinetic studies

The effect of pH on PdeM activity was determined at different values ranging from 2.0 to 10.0 at 25°C using buffers; glycine-HCl (pH 2.0–3.5), acetate (pH 4.0–5.5), sodium phosphate (pH 6.0–7.0), Tris-HCl (8.0–9.0), glycine-NaOH (pH 8.0–10.0). The optimum temperature for activity was determined by incubating the enzyme for 10 min at temperatures ranging from 15 to 75°C, with 10°C increments each in a standard reaction mixture at pH 8.5. The stability of the enzyme was evaluated by incubating the purified enzyme in a standard reaction mixture at pH 8.5 at temperatures ranging from 15 to 95°C for 15 min. This reaction mixture was cooled down to room temperature, 2 mM bis(*p*NPP) was added and reaction was carried out at 25°C for 10 min as described above.

Phosphodiesterase assay was carried out according to the modified protocol as described elsewere [10]. The reaction buffer contained 50 mM Tris-HCl (pH 8.5), 5 mM β-ME, 10 mM NaCl, 1 mM of different metal ions *viz* MnCl_2_, FeSO_4_, ZnCl_2_, NiCl_2_, MgSO_4_, CoCl_2_, 2 mM bis(*p*NPP) and 1μg PdeM Protein. EDTA (1 mM) was used to chelate metal ions. For determining the kinetic parameters, the effect of increasing Mn^2+^ ion concentration (0–6 mM) on saturation kinetics with bis(*p*NPP) and *p*NPPP as substrates was carried out in the reaction buffer described above. The Phosphodiesterase assay were performed with the same buffer system in presence of 3 mM MnCl_2_, 1 μg of protein and different concentrations of bis(*p*NPP). Reactions were initiated by adding 1 μg PdeM protein, and incubated for 10 min at 25°C with bis(*p*NPP) as substrate. A similar assay was performed with different concentrations of *p*NPPP using 2 mM MnCl_2_ as saturating concentration, whereas the protein quantity had to be increased to 5 μg as the rate of reaction was slower. The reaction was initiated by the addition of PdeM protein and incubated at 25°C for 30 min with *p*NPPP as substrate. The reactions were stopped by adding 50 mM NaOH, following which absorbance was measured at 405 nm. The molar extinction coefficient of *p*-nitrophenol was 18,450 M^-1^ cm^-1^ [31]. Specific activity was determined by quantifying *p*NP produced min^-1^ mg^-1^ protein. The kinetic parameters (K_m_ and V_max_) were determined using non-linear regression analysis by fitting the data into the Michelis-Menten equation, using GraphPad Prism (version 5.04 for Windows, GraphPad Software, San Diego, CA).

### β-Galactosidase assay

The β-galactosidase assay was performed as described elsewere [1,14]. Briefly, *E*.*coli* BL21 (DE3) harbouring pET-PdeM and *E*.*coli* BL21 (DE3) harbouring the empty pET15b vector were grown in LB medium amended with ampicillin (100 μg mL^-1^) and 3 mM Mn^+2^ ions. Cells were grown to an A_600_ in the range of 0.07 to 0.1, and 0.5 mM IPTG was added to the cultures. The cultures were grown 0.35–0.45 OD at 600 nm, and β-galactosidase activity was measured as described elsewere [32]. Expression of the recombinant enzyme was checked on 12% SDS-PAGE as described above.

### Total intrinsic fluorescence measurement for Mn^2+^ binding

The homogenously purified PdeM protein obtained after affinity purification and size-exclusion chromatography was used for studies on intrinsic fluorescence with the help of a Varian Fluorescence Spectrophotometer (Cary Eclipse) at room temperature. Fluorescence excitation was set at 280 nm, and emission spectra were recorded from 300 to 450 nm at a bandwidth of 1 nm. The excitation and emission slit widths were set at 5 nm, respectively. Each recorded spectrum represents an average of three scans. The spectra were recorded at protein concentrations of 2 μM in the presence of 50 mM Tris.Cl (pH 7.5), 50 mM NaCl and 5 mM β-ME with increasing concentration of Mn^2+^ (0–4.5 mM) or Fe^3+^ (0–2.5 mM). The background emission was eliminated by subtracting the signal of the buffer alone from the test samples. The change in intrinsic fluorescence intensity [(Fo-F)/Fo] at 340 nm with increasing metal ion concentration was plotted, and the *Kd* values were determined from a non-linear least square regression analysis of the titration data by fitting of saturation curve to one site specific binding with Hill slope using GraphPad Prism (version 5.04 for Windows, GraphPad Software, San Diego, CA). The decrease in the intrinsic fluorescence intensity of PdeM at 340 nm due to the binding of Mn^2+^ helped us to evaluate the *Kd* value for Mn^2+^ ions, the cofactors necessary for the phosphodiesterase activity by titrating the binding of increasing amounts of metal ion to a fixed concentration of the protein.

### Analysis of changes in secondary structure of PdeM by CD Spectrometry

CD data were obtained on a JASCO J815 spectropolarimeter calibrated with ammonium (+)-10-camphorsulfonate with a 2 mm path length cell in the far-UV region from 190–250 nm. Approximately, 4–8 μM protein concentration was used in 10 mM phosphate buffer (pH 7.0). The values obtained were normalized by subtracting the baseline recorded for the buffer under similar conditions. The spectropolarimeter was continuously purged with nitrogen gas before and during the experiments. All the spectra were recorded with 1 nm-spectral band width. The effect of Mn^2+^ and Fe^3+^ ions on the structure of PdeM was studied by incubating the protein in the absence and presence of different concentrations of the metal ions by measuring the ellipticity change (CD signal). Thermostability of the recombinant PdeM protein (8 μM) was monitored by measuring ellipticity change (CD signal) at 222 nm in a CD machine connected to a peltier-controlled thermostat in the temperature range of 20–80°C at every 10°C increase in the absence and presence of Mn^2+^ ions. Percentage secondary structure calculations were performed on the on-line tool SOMCD [33]. Melting temperature (Tm) defined as the temperature at which the melting profile crosses the mid-point between the two extrapolated values from the native and the denatured region of the macromolecule was obtained by fitting the data on the plateau followed by one phase decay of the GraphPad Prism software.

## Results

### Identification of gene encoding a putative phosphodiesterase from the metagenome of an Indian coalbed

In our search to find novel genes/enzymes capable of hydrolyzing phosphodiesters in the formation water of a coalbed, we determined the nucleotide sequence of the insert of a fosmid clone that hydrolyzed bis(*p*NPP). Bioinformatic analysis and annotation of the sequence of the fosmid insert revealed the presence of genes encoding a DEAD/DEAH box helicase, a glutathione S-transferase, a phosphodiesterase and an ABC transporter in an operon-like organization. The metagenomic phosphodiesterase (designated as PdeM), encoded by the metagenomic insert, showed 74%, 59% and 59% identities with the putative class III PDE from *Variovorax paradoxus* S110 (YP002946726), *D*. *acidovorans* (YP001564069) and *Acidovorax avenae* (YP004232937), respectively. However, the sequence identities of PdeM with the characterized members of class III PDEs such as CpdA of *E*. *coli* [14], PdeA of *D*. *acidovorans* [1] and Rv0805 of *M*. *tuberculosis* [15] were ≤ 20%.

BLASTp analysis with the deduced amino acid sequence of PdeM shows that it belongs to the calcineurin-like metallophophoesterases (Pfam accession ID PF00149) that shows a specific hit with a metallophosphatase domain (CD07391, E-value: 6.52e^-47^). Among the non-specific hits, the most closely related domain (TIGR04123, E-value 1.556.52e^-85^) belonged to the superfamily cI13995 that includes uncharacterized putative metallophosphoesterases associated with a DNA ligase, a helicase, and a putative exonuclease. PdeM showed a catalytic core consiting of seven conserved motifs, [D(X)H(X)_n_GD(X)_n_GNHD(X)_n_H(X)_n_GH(X)H], which are characteristic of metallophosphoesterase superfamily to which class III PDEs belong [9,10]. The metagenomic insert of the fosmid clone showing bis(*p*NPP) hydrolysis seems to have originated from a bacterium, which might belong to Comamonadaceae family, as all other genes located on this fosmid showed maximum sequence similarity to *Variovorax*, *Acidovorax* or *Comamonas* (data not shown). The gene encoding DEAD/H box helicase was also present in the vicinity of gene encoding PDE in *V*. *paradoxus* S110 (YP002946726), *D*. *acidovorans* (YP001564069) and *A*. *avenae* (YP004232937), but absent in the vicinity of genes encoding previously characterized PDEs: PdeA of *D*. *acidovorans* [1], CpdA of *E*. *coli* [14] and Rv0805 of *M*. *tuberculosis* [15].

### Phylogenetic analysis of PdeM sequence

**Fig 1:**
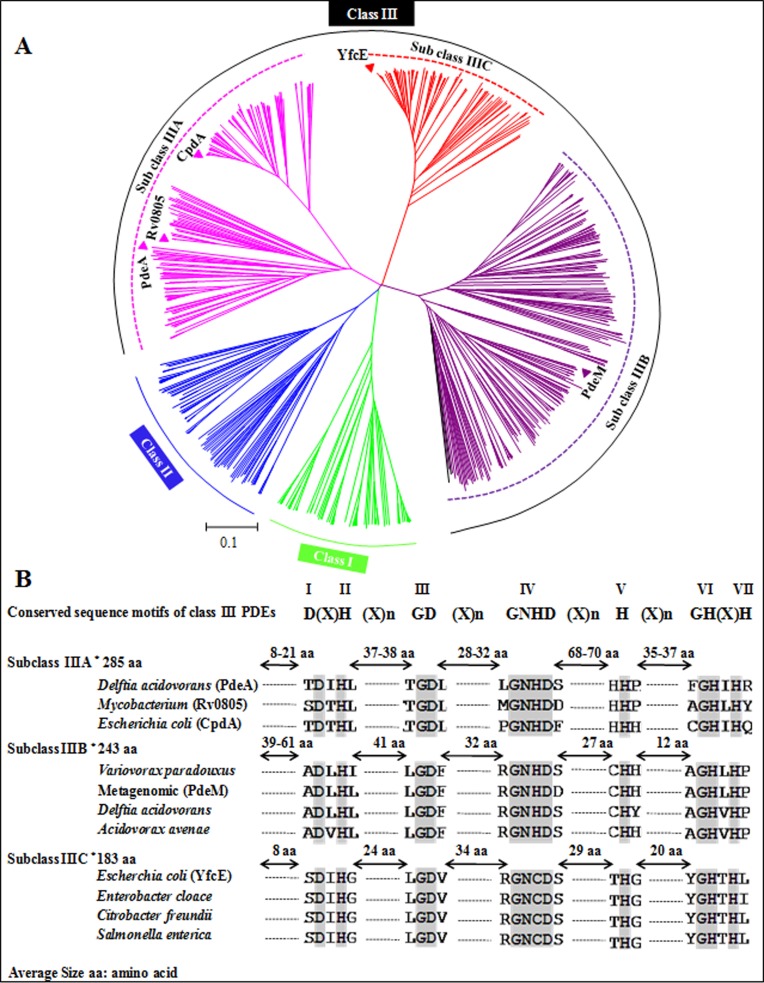
Phylogenetic relatedness of the PdeM with other phosphodiesterases. (A) Phylogenetic tree showing three distinct clades within Class III PDEs; representative members from each class were aligned and the phylogenetic tree was constructed by neighbour joining method using MEGA 5.0 software. Previously described members of Class III PDEs (Rv0805, PdeA, CpdA, and YfcE) are shown on the phylogram alongwith PdeM. (B) Comparison of the distances between conserved motifs in the members of three subclasses of Class III PDEs. The conserved sequence motifs and their numbers are indicated on the top and are also as well as highlighted in grey in the sequences. Average size (number of amino acids) of each subclass is also indicated.

In order to decipher phylogenetic relatedness of PdeM, a neighbour-joining tree was constructed from the multiple sequence alignment of 647 randomly selected phosphodiesterase sequences including the previously characterized PDEs (Fig. 1A). The phylogram showed 5 broad clades, one of Class I (represented by 79 homologs) and the second of Class II PDEs (represented by 82 homologs). Class I and Class II PDEs are characterized by H(X)_3_H(X)_25–35_D/E and H(X)HLDH motifs, respectively, that are involved in the binding of metal ions in the catalytic domain [9].

In addition to these two classes, we observed three other clades that harboured the characteristic [D(X)H(X)_n_GD(X)_n_GNHD(X)_n_H(X)_n_GH(X)H] motifs of the class III PDEs [10]; one consisting of 178 homologs, the second with 208 homologs, and the third with 100 homologs having a GNCD motif, instead of a GNHD, as the IVth motif in the characteristic signature motifs of class III PDEs. On the basis of size and sequence of the PDEs, we now designate these three clades as three subclasses of class III PDEs: subclass A represented by Rv805, CpdA and PdeA, subclass B represented by PdeM, and subclass C represented by YfcE [27]. The average size of the proteins in the subclass IIIA, IIIB and IIIC was about 285 aa, 243 aa and 183 aa, respectively. Although PDEs of the three subclasses of class III PDEs show occurrence of 7 conserved motifs, the distances between them are characteristically different in each subclass (Fig. 1B). The distinguishing differences among the three subclasses were seen between motif II (H) and III (GD), motif IV (GNH/CD) and V (H), and motif V (H) and VI (GH). Thus, based on differences in spacing between the conserved motifs, we propose three distinct subclasses within class III PDEs, and show that PdeM represents a new subclass (IIIB) of the class III PDEs to be characterized; all other proteins of this subclass are uncharacterized bacterial and archaeal proteins.

### Overexpression and purification of PdeM

Overexpression and purification of the recombinant PdeM yielded 7–10 mg purified enzyme per litre culture. The theoretical molecular mass of PdeM without and with His-tag was ~27.3 kDa and 29.3 kDa, respectively. Resolution of the extracted protein in SDS-PAGE showed a single protein of ~29.3 kDa indicating the apparent purity of extracted protein. The affinity purified protein was used for further assays and characterization.

### PdeM is a metal dependent enzyme

**Fig 2:**
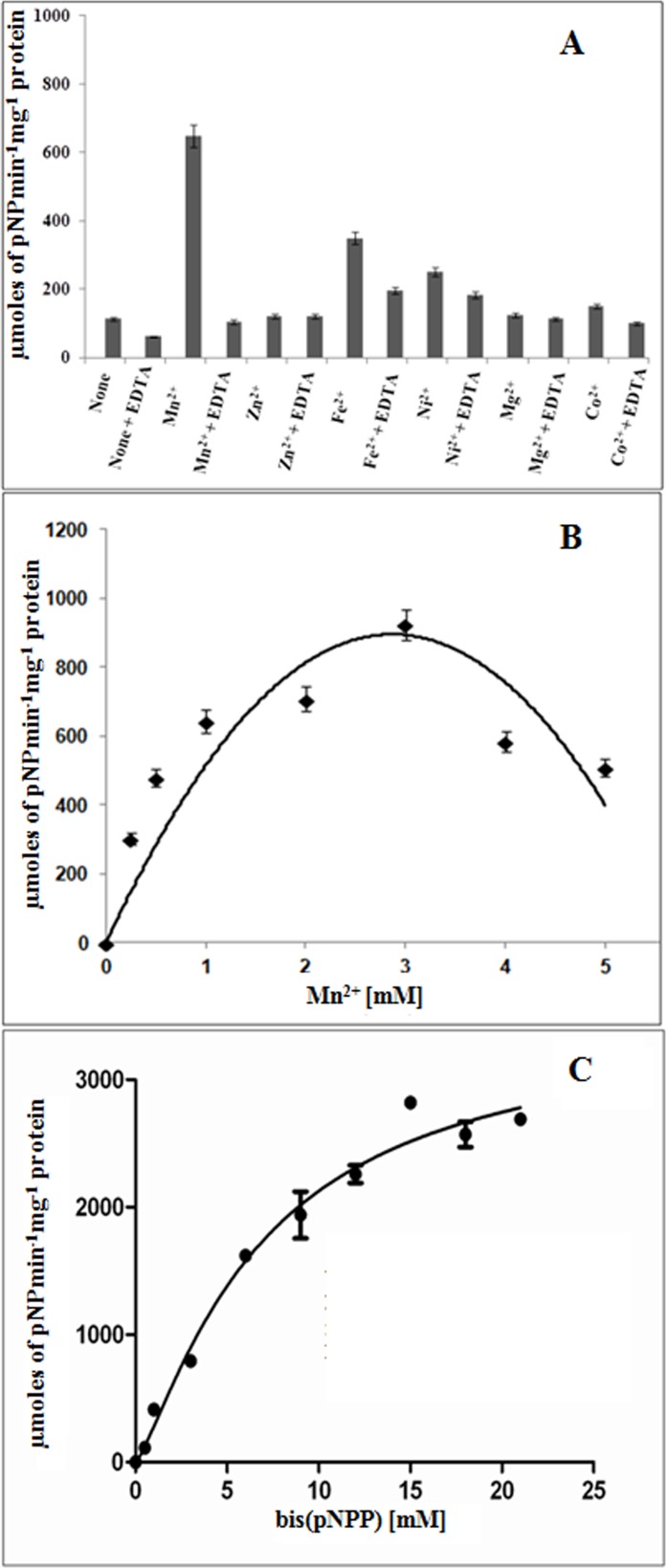
PdeM is a metal dependent enzyme. (A) Effect of different metal ions on the phosphodiesterase activity of PdeM. The metals as well as EDTA were used at 1 mM concentration for all reactions. (B) Effect of different concentrations of Mn^2+^ on the phosphodiesterase activity of PdeM. The assays were performed using 2 mM bis(*p*NPP) as substrate and 1μg of enzyme. (C) Effect of different concentrations of bis(*p*NPP) on the phosphodiesterase activity of PdeM. Reactions were performed in presence of 3 mM Mn^2+^ and 1μg of enzyme.

Since phosphodiesterases are known to be metal dependent enzymes, we studied the effect of divalent metal ions such as Mn^2+^, Zn^2+^, Fe^2+^, Ni^2+^, Mg^2+^, and Co^2+^ on the ability of PdeM to hydrolyze bis(pNPP) under standard reaction conditions containing metal ions with or without 1 mM EDTA. PdeM showed maximum activity at pH 8.5 in presence of 1 mM Mn^2+^ ion (648 μmoles p-nitrophenol produced min^-1^ mg^-1^) whereas in the absence of Mn^2+^ no activity was detected (Fig. 2A). Addition of 1 mM EDTA in Mn^2+^ coupled reaction inhibited the PdeM activity by ~6 fold (105 μmoles min^-1^ mg^-1^), reflecting that sequestration of Mn^2+^ ions by EDTA was responsible for the inhibition of the enzymatic activity, and that Mn^2+^ ions were required for the PDE activity. PdeM exhibited maximum activity (923 μmoles min^-1^ mg^-1^) at 3 mM Mn^2+^ concentration indicating a requirement of relatively high amount of metal ions for its optimal activity (Fig. 2B). Similarly, treatment with EDTA also reduced the PdeM activity by 50% and 30% in case of Fe^2+^ and Ni^2+^, respectively.

### PdeM is a phosphodiesterase having phosphonate-ester hydrolase and nuclease activities

We screened the ability of PdeM to hydrolyze *p*-nitrophenyl phosphate (*p*NPP, a monoester), bis(*p*NPP) (a diester) and methyl parathion (a phosphotriester). Since PdeM failed to hydrolyze *p*NPP or methyl parathion under any condition it indicated that it lacks phosphomonoesterase and phosphotriesterase activities (data not shown). The ability of PdeM to hydrolyze bis(*p*NPP) as well as *p*-nitrophenyl phenylphosphonate (*p*NPPP), however, indicated that it was a phosphodiesterase with phosphonate ester hydrolase activity. The inability of PdeM to hydrolyze *p-*nitrophenyl phosphoryl choline (*p*NPPC), thymidine 5’-monophosphate *p*-nitrophenyl ester (*p*NPTm), cAMP and cGMP indicated that PdeM is capable of hydrolyzing aromatic phosphate compounds but does not act on cAMP, cGMP and other phosphodiesters.

**Fig 3:**
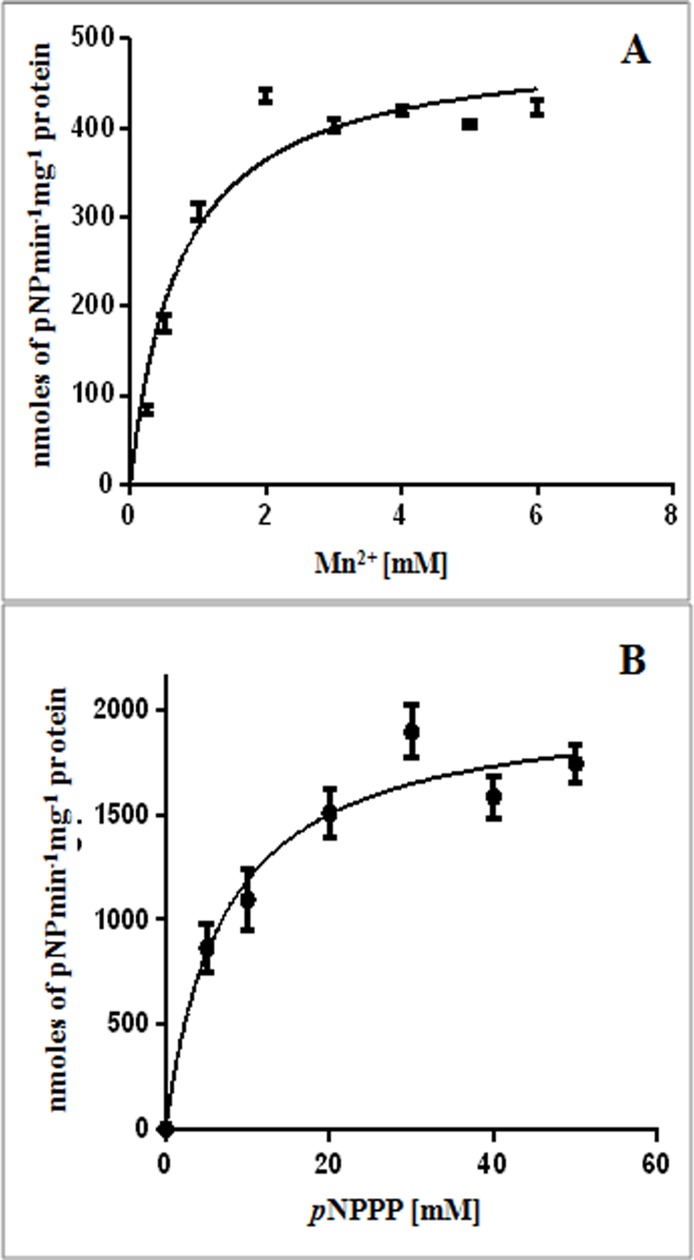
Phosphonate-ester hydrolase activity of PdeM. (A) Effect of Mn^2+^ ion concentrations on the phosphonate-ester hydrolase activity of PdeM. The assays were performed using 2 mM *p*NPPP as substrate and 5 μg of enzyme. (B) Effect of increasing concentration of *p*NPPP on the phosphoesterase activity of PdeM. Reactions were performed in 1 mL reaction volume containing 2 mM Mn^2+^ and 5 μg of enzyme.

Michaelis-Menten kinetics of PdeM with bis(*p*NPP) as substrate exhibited *K*_*m*_, *V*_*max*,_ *K*_*cat*_, and *K*_*cat*_/*K*_*m*_ values of 10.21 ± 1.6 mM, 3473 ± 373.8 μmoles min^-1^ mg^-1^, 102.45x10^3^ min^-1^ and 100.34x10^5^ M^-1^ min^-1^, respectively (Fig. 2C). Like YfcE of *E*. *coli* [27] and several eukaryotic PDEs [34] PdeM shows very low affinity for bis(*p*NPP). But unlike most other PDEs, it shows low affinity for Mn^2+^. PdeM showed Mn^2+^ ion-dependent phosphonate ester hydrolase activity, which reached its maximum at 2 mM Mn^2+^ (Fig. 3A). The kinetics of reaction of PdeM with increasing concentrations of *p*NPPP as substrate in presence of 2 mM Mn^2+^ showed *K*_*m*_, *V*_*max*,_ *K*_*cat*_, and *K*_*cat*_/*K*_*m*_ values of 7.2 ± 1.4 mM, 2041 nmoles min^-1^ mg^-1^, 60.21 min^-1^ and 8.3x10^3^ M^-1^ min^-1^, respectively (Fig. 3B). The activity of PdeM decreased by ~8 fold at 95°C (53 μmoles min^-1^ mg^-1^) in comparison to 25°C (416 μmoles min^-1^ mg^-1^), indicating the thermolabile nature of PdeM protein.

In an attempt to identify a natural substrate for PdeM, the activity of PdeM against single stranded DNA, linear DNA, genomic DNA, plasmid DNA as well as RNA was investigated. PdeM showed Mn^2+^-dependent nuclease activity against, double stranded linear DNA, plasmid DNA and genomic DNA. However, no nuclease activity was observed with single stranded DNA and RNA as shown in S4 Fig.

### PdeM does not hydrolyze cAMP *in vivo* and *in vitro*

**Fig 4:**
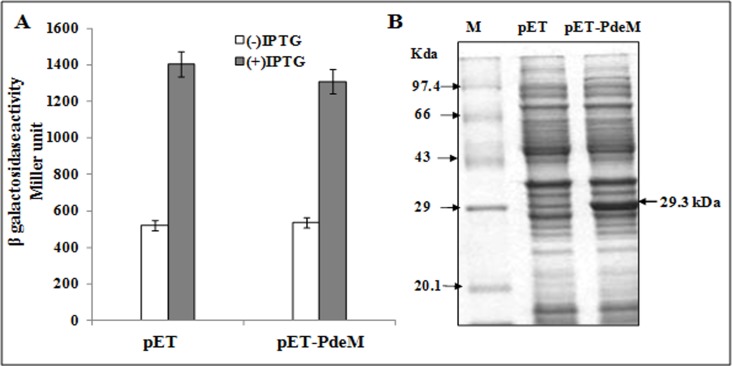
*In vivo* assay for cAMP phosphodiesterase acivity of PdeM. (A) β-Galactosidase activity of *E coli* BL21 (DE3) harbouring pET15b or its derivative pET-PdeM without or with induction by IPTG. (B) SDS-PAGE of the total proteins extracted from *E coli* BL21 (DE3) harbouring pET15b or its derivative pET-PdeM without or with induction by 0.5 mM IPTG. Lane M: protein molecular weight marker; lane pET: induced cells of *E coli* BL21 (DE3) harbouring pET15b, and lane pET-PdeM: induced cells of *E*. *coli* BL21 (DE3) harbouring pET-PdeM.

Since all the class III phosphodiesterases, characterized so far, exhibit the ability to hydrolyze cAMP *in vivo*, we carried out an *in vivo* assay to test if PdeM could also hydrolyze cAMP *in vivo*. The gene encoding β-galactosidase is under catabolite repression in glucose containing medium. Under glucose deficiency, the β-galactosidase expression is upregulated due to the synthesis of cAMP, which is required by the positive regulator protein, CAP, to express *lacZYA* operon. Overexpression of PdeM in *E*. *coli* is expected to prevent intracellular accumulation of cAMP leading to the expression of *lacZYA* operon in *E*. *coli*, if cAMP is a substrate for PdeM. For this, we compared the β-galactosidase activity of *E*. *coli* BL21 (DE3) cells harbouring pET-PdeM and *E*.*coli* BL21 (DE3) harbouring the empty pET15b vector. Both, *E*.*coli* BL21 (DE3) harbouring pET-PdeM and *E*.*coli* BL21 (DE3) harbouring the empty pET15b vector were grown in LB medium amended with ampicillin (100 μg mL^-1^) and 3 mM Mn^+2^. No significant difference was observed between the β-galactosidase activities of the IPTG-induced (0.5 mM) culture harbouring or lacking PdeM expressing plasmid. Addition of IPTG (analogue of lactose) increased β-galactosidase activity in *E*. *coli* BL21(DE3) cells harbouring *pde*M cloned in pET15b as well as in *E*. *coli* BL21(DE3) harbouring pET15b (Fig. 4A). In order to ensure that PdeM protein was expressed in *E*.*coli* BL21 (DE3) cells harbouring *pde*M in pET15b, we also analyzed the total proteins in SDS-PAGE which indicated synthesis of the recombinant PdeM protein (Fig. 4B). Despite the synthesis of PdeM protein in *E*. *coli*, no significant decline in β-galactosidase activity indicated that PdeM was not able to hydrolyze cAMP. The inability of recombinant PdeM protein to hydrolyze cAMP and cGMP *in vitro* (as described in Materials and Methods) also indicated that PdeM lacks the ability to hydrolyze cNMPs. Hence, unlike the members of subclass III A (CpdA, PdeA and Rv0805), which show hydrolysis of cAMP; PdeM lacks the ability to hydrolyze intracellular cAMP.

### Metals do not affect oligomerization of PdeM

Since Mn^2+^ and Fe^3+^ ions are known to affect the oligomeric status of Rv0805, we examined the effect of these metal ions on the oligomeric status of PdeM. FPLC analysis of the purified protein extracted from the cultures grown in presence and absence of metal ions (MnCl_2_, FeCl_3_ or both) showed a major peak at ~17 mL elution volume corresponding to ~29.3 kDa as compared to the standard marker proteins. This observation suggests that PdeM exists predominantly as a monomer in solution and unlike Rv0805, the addition of Mn^2+^ and Fe^3+^ ions or both did not affect the monomeric nature of the protein. PdeM retained its monomeric form even at higher concentration of Mn^2+^ (3 mM; data not shown).

**Fig 5:**
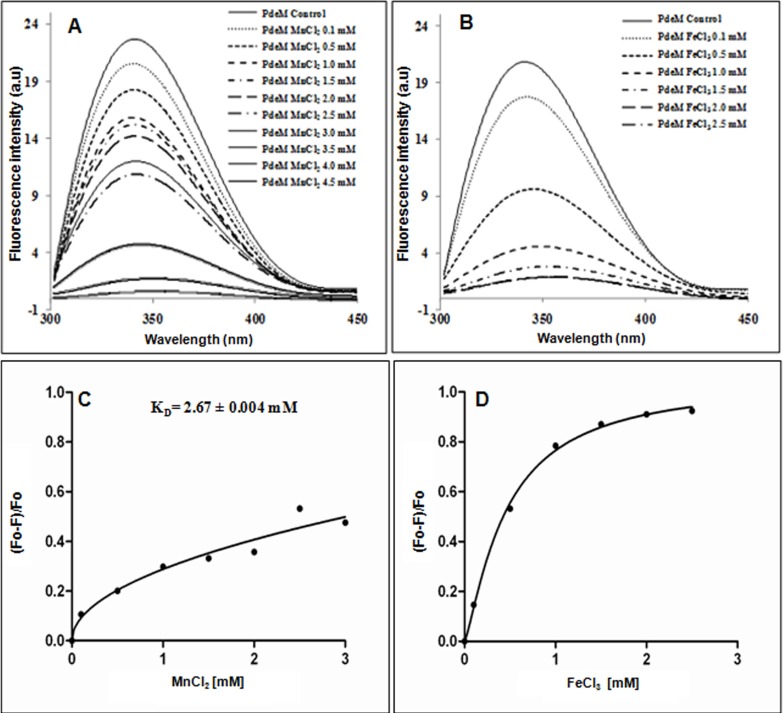
Effect of metal ions on the intrinsic fluorescence of PdeM. (A) Effect of Mn^2+^ and, (B) Effect of Effect of Fe^3+^ concentrations on the intrinsic tryptophan fluorescence of the purified PdeM protein. Saturation curves were generated from the intrinsic fluorescence data by plotting the change in fluorescence intensity at 340 nm as a function of increasing concentrations of (C) Mn^2+^ and (D) Fe^3+^.

PdeM contains 7 tryptophan residues which are evenly distributed in the protein. Consequently, PdeM shows a fluorescence emission maximum at 340 nm. Increasing the concentration of Mn^2+^ (0.1 mM to 3.0 mM) led to a gradual decline in the fluorescence upto 53% of the basal level (Fig. 5A). However, a further increase in Mn^2+^ from 3.0 mM to 4.5 mM led to a drastic reduction in its fluorescence upto 98%. This observation suggested that an increase in Mn^2+^ concentration upto 3 mM induced a favorable change in the conformation of PdeM protein which increased the enzyme activity whereas further increase in Mn^2+^ concentration abruptly decreased the fluorescence intensity of the protein (Fig. 5A). In range of 2–3 mM of Mn^+2^ ion concentrations there is a change in the secondary structure of PdeM, which might result in a change in the tertiary structure as evident by abrupt decrease in fluorescence intensity beyond 3 mM Mn^2+^. Hence, conformation beyond 3 mM Mn^+2^ ions may not be favourable for the optimum activity of PdeM. This observation corroborates well with our metal saturation kinetic studies, where maximum phosphodiesterase activity of PdeM was observed at 3 mM Mn^2+^ and further increase of Mn^2+^ reduced the PdeM activity (Fig. 2B). In case of Fe^3+^, the emission declined by 53% even at 0.5 mM concentration while at 1 mM it was reduced by 78% (Fig. 5B). Also, the lack of phosphodiesterase activity in presence of 1 mM Fecl_3_ (data not shown) is in agreement with our fluorescence data in presence of varying concentrations of Fe^3+^.

Although an increase in Mn^2+^ concentration decreased the fluorescence intensity, neither emission maximum (340 nm) nor spectral bandwidth was affected. A saturation curve was generated by plotting the change in fluorescence intensity at 340 nm as a function of added MnCl_2_ (Fig. 5C). The *Kd* value of 2.67±0.004 mM was estimated for Mn^2+^ to one site specific binding with Hill Slope. Approximately 53% of the intrinsic fluorescence was accessible to the quencher Mn^2+^ ion upto 3 mM concentration. Analysis of the Hill plot generated from the Mn^2+^ ion binding data yielded a Hill coefficient of 0.5, indicating negative cooperativity. A drastic decline in the fluorescence intensity at 340 nm due to increase in FeCl_3_ concentration indicated complete denaturation of PdeM hence no *Kd* value was calculated (Fig. 5D).

### Mn^2+^ and Fe^3+^ increases the α-helical content of PdeM protein

**Fig 6:**
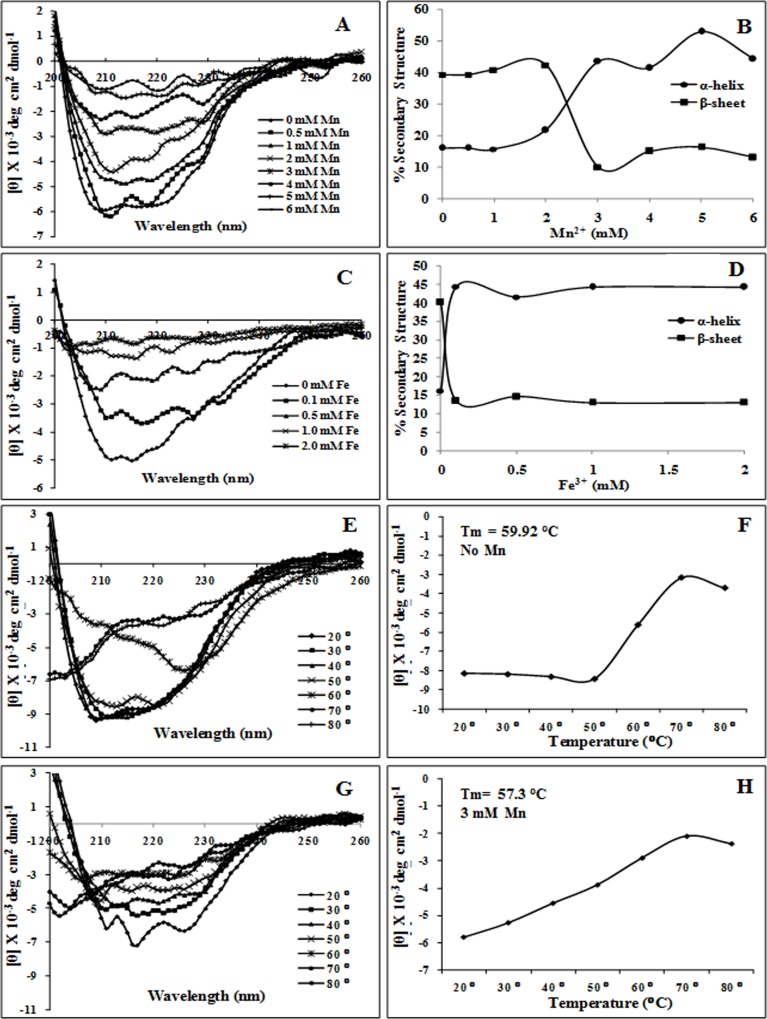
Effect of metal ions on the secondary structure of PdeM. (A) Effect of increasing Mn^2+^ ions on the Circular Dichroism (CD) spectra of PdeM recorded in the far-UV region (from 200 to 260 nm) at 25°C at a protein concentration of 5 μM in 10 mM phosphate buffer (pH 7.0). (B) Effect of increasing concentrations of Mn^2+^ ions on the secondary structural elements (α-helix and β-sheets). (C) Effect of increasing concentrations of Fe^3+^ ions on the CD spectra of PdeM protein recorded in the far-UV region at a concentration of 4.11 μM PdeM in 10mM phosphate buffer (pH 7.0). (D) Effect of increasing concentrations of Fe^3+^ ions on the secondary structural elements (α-helix and β-sheets). The spectra reported are representative from of the three independent experiments. (E) Effect of temperature on the ellipticity of PdeM analyzed by CD spectroscopy in the absence of Mn^2+^ ions. (F) The thermal melting profile of PdeM monitored in the absence of Mn^2+^ ions by CD signals at 222 nm. (G) Effect of temperature on the ellipticity of PdeM analyzed by CD spectroscopy in the presence of 3 mM Mn^2+^ ions. (H) The thermal melting profile of PdeM monitored in the presence of 3 mM Mn^2+^ ions by CD signals at 222 nm. The temperature of the enzyme solution (8 μM) in the cell was gradually increased in a step-wise manner from 20°C to 80°C. The CD signals were recorded after equilibrating the cell for at least 5 min, after each desired temperature was attained.

On the basis of circular dichroism (CD) spectra, we analyzed changes in secondary structural components of PdeM that occur at different concentrations of Mn^2+^ and Fe^3+^ ions. CD spectrum for the native PdeM protein showed negative ellipticities, with a double minimum at 208 nm and 222 nm, typical of α-helical content of the proteins (Fig. 6A). The SOMCD analysis revealed that the native PdeM contained approximately 16% α-helical content, 39.2% β-sheets, 35% random coils and 9.7% turns. This secondary structural composition remained almost constant upto 1 mM Mn^2+^. At 2 mM Mn^2+^, PdeM showed 21.7% α-helical content, 42.3% β-sheets, 26.8% random coils and 9.2% turns. A major structural transition occured upon increasing the Mn^2+^ concentration to 3 mM resulting in an increase in the α-helical content to 43.5%, and a decrease in the β-sheets to 10% alongwith minor changes in random coils and turns (Fig. 6B). On increasing the Mn^2+^ ion concentration further from 4 mM to 6 mM, the secondary structural composition of PdeM remained almost constant. An approximate increase of 27.5% in the α-helical content, and a decrease of 29.2% in the β-sheets from 0 to 3 mM Mn^2+^ ion concentration indicate a major structural interconversion of β-sheets to α-helices due to the binding of Mn^2+^ ions to PdeM (Fig. 6B). The addition of Fe^3+^ ion to 0.1 mM concentration led to a drastic increase in the α-helical content to 44.4%, and a decrease in the β-sheets to 13.4% with minor changes in random coils and turns in the PdeM protein (Fig. 6C). Further increase in the Fe^3+^ concentration upto 2 mM did not seem to affect the secondary structure of PdeM any further (Fig. 6D). The drastic change in the secondary structure of PdeM, which occurred at 0.1 mM Fe^3+^ itself seems to be enough for the loss in the activity.

We also studied the effect of incubation temperature on the secondary structural composition of PdeM in the absence or presence of 3 mM Mn^2+^ in the range of 20°C to 80°C. The prominent features of the helix related CD spectrum remained unperturbed from 20°C to 50°C. However, above 50°C, the amplitudes of the CD spectral bands decreased sharply; and at 70°C, the spectrum observed was consistent with that of a largely unfolded polypeptide chain (Fig. 6E). The melting profile of PdeM was determined by continuously monitoring mean residue ellipticity [θ] at 222 nm in the temperature range of 20°C-80°C. The ellipticity showed a sharp thermal transition in the range of 50°C-70°C (Fig. 6F). The effect of temperature on the melting behaviour of PdeM in the presence of 3 mM Mn^2+^ shows that there was a gradual decline in the α-helical content from 20°C to 70°C (Fig. 6G), which is in sharp contrast to that obtained in the absence of Mn^2+^ (Fig. 6H). The Tm of PdeM in the absence and presence of 3mM Mn^2+^ was 59.92°C and 57.3°C, respectively (Fig. 6F, H) showing an increase of 2.6°C in the absence of Mn^2+^. This indicated that the binding of Mn^2+^ ions to PdeM led to a minor loss in the stability of the PdeM protein.

### Homology modelling and structural analysis of PdeM

For modelling the structure of PdeM, we found three proteins in the Protein Data Bank, YfcE of *E*.*coli* (PDB ID: 1SUL1_A, 22% identity, E-value = 3.3e^-20^), Rv0805 of *M*. *tuberculosis* (PDB ID: 3IB7_A, 23% identity, E-value = 2.4e^-15^) and MJ0936 of *Methanococcus jannaschii* (PDB ID: 1S3L_A, 13% identity, E-value = 9.6e^-20^), which showed good alignment of the predicted secondary structural features with PdeM, but low sequence similarity. Utilizing the structural features of all the three proteins, we constructed a homology model of PdeM. The Ramachandran plot of this model showed lowest energy, and 94% of all residues were in the allowed region. The root mean square deviations (RMSD) of the modelled structure of PdeM from the template structures, 1SUL_A (154 Cα), 1S3L_A (128 Cα), 3IB7_A (123 Cα) were 1.878 Å, 2.090 Å and 2.479Å, respectively. RMSD was evaluated on the superposition across Carbon-α atoms of 154, 128 and 123 residues of 1SU1_A, 1S3L_A, 3IB8_A respectively, over PdeM.

**Fig 7:**
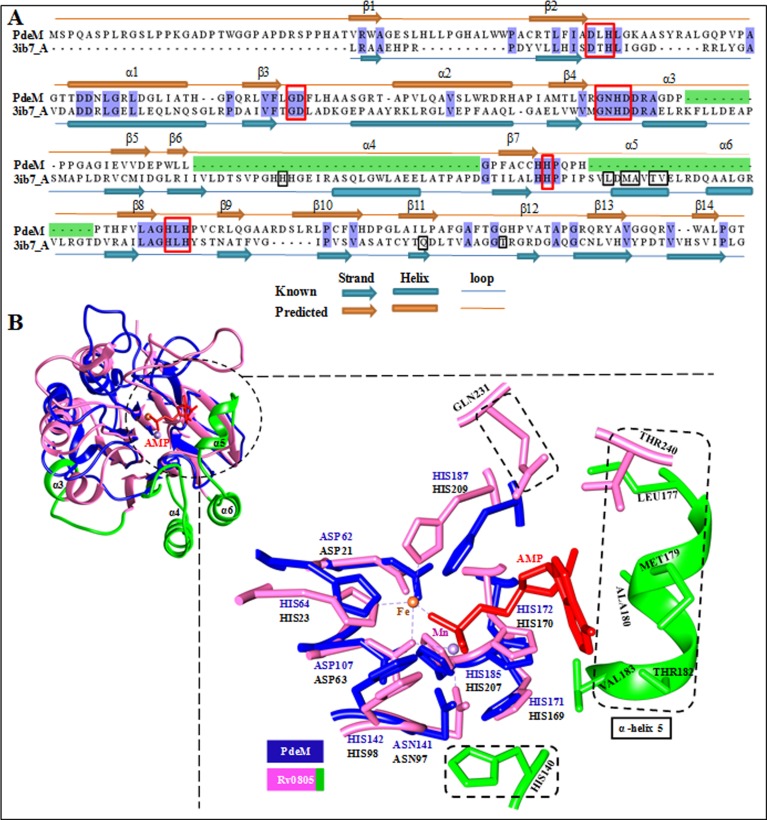
Comparison of the sequence and structure of PdeM with Rv0805. (A) Pairwise sequence alignment between the full length cyclic AMP phosphodiesterase Rv0805 of *M*. *tuberculosis* (PDB ID: 3IB7) and PdeM. The annotation of secondary structure is displayed at the top. Identical residues in the alignment are shaded in blue. Regions which are missing in PdeM are shaded in green. All motifs with conserved residues coordinating the metal ions are marked in square bracket (red outline). Residues interacting with the AMP, cAMP and bis(*p*NPP) are marked in square bracket (black outline). (B) Superimposition of the homology model structure of PdeM (colored as blue) and template Rv0508 structure (colored as pink), and stereoview of the superimposition of active site residues in the homology model of PdeM (colored as blue) and the template structure of Rv0805 (PDB ID: 3IB8; colored as pink). Bound metals/ions are represented as a sphere model. Residues within 5Å interaction zone of metals are shown as sticks. Residues interacting with AMP (3IB8_A), cAMP and bis(*p*NPP) are enclosed in broken line square bracket. Regions of Rv0805 corresponding to the missing regions in PdeM are shown in green color. Structure visualization and analysis was performed using Chimera tool.

Rv0805 is the first member of the Class III PDEs to be characterized biochemically, computationally and structurally [10, 15]. It is the only member of Class III PDEs whose 3D structure bound to its ligands is known, and to which PdeM shows maximum structural similarity. Critical comparison of the sequence and modelled structure of PdeM with that of Rv0805 revealed interesting similarities and differences, which could explain some of the experimental observations related to the function of PdeM. The fold and overall structure of PdeM are very similar to that of Rv0805 (Fig. 7A). However, PdeM showed two significant deletions: one of 33 residues including α-helix 4, located between β6-β7 regions of Rv0805, and the second, of 24 residues which include α-helices 5 and 6, located between β7-β8 regions of Rv0805 (Fig. 7B). Although both these deletions in PdeM are away from the substrate-binding site, they seem to affect the affinity of PdeM for Mn^2+^ and also the catalytic activity. They seem to have altered the catalytic site, making it wide open to accommodate substrates larger than those accomodated by Rv0805. The most striking structural feature of PdeM is the deletion of residues corresponding to Met179, Ala180, Thr182 and Val183 of the helix 5, and preceding loop residues Leu177, along with Gln231 and Thr240 of the loop between β11- β12 region in Rv0805, which interact and hold the sugar and base moieties of cAMP (Fig. 7B). All these residues are also involved in the interaction of Rv0805 with bis(*p*NPP). Lack of these residues in PdeM suggests a low affinity for cAMP or cGMP (2′, 3′ or 3′, 5′ forms), and consequent lack of phosphodiesterase activity on these substrates. The inability of PdeM to hydrolyze cAMP can also be explained by the absence of residue corresponding to His140 of Rv0805, which is thought to be involved in the binding of cAMP [10], as His140Ala mutant lost the ability to hydrolyze cAMP completely. The low affinity (K_m_ 10.21 mM) of PdeM for bis(*p*NPP) might be due to the absence of His140, which was shown to be responsible for the high affinity of Rv0805 towards bis(*p*NPP) as the K_m_ of His140Ala mutant of Rv0805 for bis(*p*NPP) declined to 1.3 mM from 9 mM [10]. His140 in Rv0805 is placed in such a way that it is able to create a gate at the metal binding site. Lack of His140-like residue in PdeM at such a position may also lower its affinity for Mn^2+^.

## Discussion

In this study we discovered a novel phosphodiesterase (PdeM), from the metagenome of formation water of an Indian coalbed, which seems to have originated from a bacterium, phylogenetically related to *Variovorax*, *Acidovorax* or *Comamonas* genera of the Comamonadaceae family of β-proteobacteria. Occurrence of *Acidovorax and Comamonas* in the microbial communities present in the formation water of the same coalbed was earlier suggested by our previous study showing abundance of 16S rRNA sequences showing phylogenetic relatedness to *Acidovorax* and *Comamonas* [19]. Not only does PdeM show a limited sequence identity (20–30%) with the characterized members of Class III PDEs, it also shows considerable structural, biochemical and biophysical differences as well. Our study reveals that PdeM is a Mn^2+^ phosphodiesterase with phosphonate-ester hydrolase and nuclease activities, which is active as monomer, and does not oligomerize by increasing Mn^2+^ concentration. It does not hydrolyze phosphomonoesters, phosphotristers, cAMP, cGMP, single stranded DNA, RNA, *p*NP-TMP and *p*NPPC. Like several eukaryotic PDEs [34] and YfcE of E coli [27], PdeM shows very low affinity for bis(*p*NPP). But unlike most other PDEs, it shows low affinity for Mn^2+^. Although it requires high levels of Mn^2+^ for optimal PDE activity, an increase beyond 3 mM alters the secondary structural components of PdeM so that its activity is adversely affected.

When we aligned and compared CpdA, PdeA, YfcE and Rv0805 and other homologs in class III PDEs alongwith PdeM, we observed that they made three distinct subclasses: CpdA, PdeA and Rv0805 are grouped in Subclass A, YfcE represents Subclass C, and PdeM is the member of the Subclass B, which is being described for the first time in this study. The differences in distances between the 7 conserved motifs are due to characteristic deletions of amino acid residues in Subclass B and Subclass C. These deletions are thought to affect the activity of PDEs of each subclass as a consequence of change in their affinity for substrates and metals.

Members of the subclass IIIA exist as dimer (Rv0805) or trimer (PdeA). Some members of the metallophosphoesterase superfamily (for example, MPPED2 and LMO2642), however, have also been reported to be monomeric [35, 36]. Unlike Rv0805, which exists predominantly in the dimeric form, and addition of extra Mn^2+^ and Fe^3+^ ions renders them almost entirely dimeric [10], PdeM is monomeric and does not oligomerize in the presence of Mn^2+^ or Fe^3+^. The dimerization of Rv0805 is mediated by hydrogen bonds between main chain atoms of residues Ser211 and Asn213 of β9 strand and His261 and Val 263 of β14 of the two PDE monomers [10]. The residues corresponding to Ser211, Asn213 and Val 263 of Rv0805 were replaced by Val, Arg and Ala, respectively, in PdeM, and the residue corresponding to His261 was missing. Moreover, β9 of PdeM retracts and adopts a conformation which is different from β9 of Rv0805. The attainment of such a conformational specialization is due to the loop connecting β9 and β10, which adopts a sharp turn in Rv0805, and with an insertion of 5 residues confer a large loop in PdeM. In addition, W243 with its bulky side chain protrudes at the dimer forming surface in PdeM, which is likely to restrict dimerization, as shown in S7 Fig.

YfcE is the only characterized member of the subclass C [27], in which the members are of the average size of 183 aa, and have a characteristic GNCD instead of GNHD as the IVth motif, whereas the other 6 motifs were identical to the signature motifs of Class III PDEs. YfcE is a tetrameric protein which shows Mn^2+^ dependent hydrolysis of bis(*p*NPP) with high affinity for Mn^2+^, but very low affinity for bis(*p*NPP). Although it can also hydrolyze *p*NP-TMP and *p*NPPC, it failed to hydrolyze phosphomonoesters, phosphotriesters, cAMP, cGMP, single- or double stranded DNA. Similar to YfcE protein, PdeM does not exhibits activity against phosphomonoesters, phosphotriesters, cAMP, cGMP, single stranded DNA and RNA. Comparative modelling of PdeM suggests that overall structures of PdeM and YfcE are quite similar (RMSD 1.88 Å), yet structural specialization in PdeM, in terms of substrate binding site, makes it distinct from the other two subclasses.

The Class III PDEs belong to a large family of metal dependent phosphodiesterases which contain two metals; Fe^2+^, Mn^2+^, Ni^2+^ or Zn^2+^ at site 1 and Fe^2+^, Zn^2+^ or Mn^2+^ at site 2 [9]. The divalent cations increase the activity of enzyme by assisting in the folding of the expressed enzyme [37]. The requirement of PdeM for metals was quite different from that of Class III PDEs. The catalytic activity of PdeM was enhanced maximally by Mn^2+^, and also by Fe^2+^ and Ni^2+^, but Mg^2+^, Zn^2+^, and Co^2+^ had negligible effect on its activity. The higher *Kd* value for Mn^2+^ binding to PdeM (2.67±0.004 mM) as compared to that of YfcE (5.56±0.06 μM) and Rv0805 (21±5 μM) indicated a considerably lower affinity of binding of Mn^2+^ to PdeM. This is in agreement with our data which shows ~6 fold loss in the phosphodiesterase activity of PdeM in the presence of only 1 mM EDTA, contrary to Rv0805 where Mn^2+^ and Fe^3+^ remain bound to the protein even after treatment with 10 mM EDTA.

While studying the effect of Mn^2+^ on the activity of PdeM, we noted that PdeM shows optimal activity at 3 mM Mn^2+^ but a further increase in its concentration had an adverse effect on its activity. It may be due to the changes in the tertiary structure of PdeM that affect conformation as well as activity of the PdeM [38]. Changes in the total intrinsic fluorescence of proteins upon their interaction with metals have been used to derive information about changes in their tertiary structures [39, 40]. On the basis of the effects of Mn^2+^ and Fe^3+^ on the total intrinsic fluorescence of the 7 tryptophan residues present in PdeM, we anticipated changes in the secondary structure of PdeM, which around 3 mM Mn^2+^, favour optimal activity. But, higher concentrations of Mn^2+^ lead to extensive alterations in the structure of PdeM that impact its activity adversely. We validated this observation by studying secondary structural elements in PdeM using CD spectroscopy. It was noted that an increase in Mn^2+^ concentration led to an increase in the α-helical content of PdeM, which acquires the required structural features for optimal activity on bis(*p*NPP) as substrate at 3 mM Mn^2+^ concentration. But, above 3 mM Mn^2+^, PdeM seems to acquire a secondary structure that is not optimum for its enzymatic activity. Class I PDEs contain an entirely α-helical catalytic domain that co-ordinates two metals involved in cNMP hydrolysis, whereas Rv0805 is a βαβαβ protein with a shallow groove in which cNMP binds. Non-cyclic phosphodiesters bind at sites other than those involved in the binding of cyclic phosphodiesters [10]. However, PdeM has considerably lower α-helical content, and an increase in the α-helical content as well as activity of PdeM with an increase in Mn^2+^ concentration suggests that α-helices may be required for the optimal activity of PDEs.

This study has led to the division of Class III PDEs into 3 distinct subclasses; PdeM, the phosphodiesterase isolated from the coalbed metagenome, being the first characterized member of the subclass IIIB. Unlike the members of subclass A, the members of subclasse B (PdeM) and subclass C (YfcE) do not hydrolyze cNMPs. We do not yet know the actual physiological substrates that are hydrolyzed by the members of subclass IIIB and IIIC. Both of these subclasses have high structural and functional similarity, seem to act on linear rather than cyclic phosphodiesters and may require a cellular binding partner for the activation and recognition of the substrate as normally done by multi-domain phosphatases [27].

## Supporting Information

S1 Table Accession numbers of sequences used for studying the phylogenetic relationship of PdeM with other phosphodiesterases. (DOC)

S1 Fig Comparison of the organization of phosphodiesterase encoding gene of the metagenomic clone with those from other bacterial genomes including previously characterized members of class III phosphodiesterases. (TIF)

S2 Fig Overexpression and purification of PdeM protein. (A) 12% SDS-PAGE depicting overexpression. (B) Ni-NTA affinity-purification of PdeM extracted from the *E*. *coli* BL21 (DE3) grown in the absence or presence of 100 μM Mn^2+^, Fe^3+^ or both. U- Uninduced cells of *E*. *coli* BL21 (DE3); C- *E*. *coli* BL21 (DE3) harbouring the pET-PdeM induced with 0.5 mM IPTG at 16°C for 20 hour; Mn- PdeM overexpressed in presence of 100 μM Mn^2+^; Fe- PdeM overexpressed in presence of 100 μM Fe^3+^; Mix- PdeM overexpressed in presence of 100 μM of Mn^2+^ and Fe^3+^ each, and M- Protein molecular size marker (14–97.4 kDa).
(TIF)

S3 Fig Effect of temperature on phoshodiesterase activity of PdeM. (TIF)

S4 Fig Mn^2+-^dependent nuclease activity of the PdeM. PdeM showed nuclease activity against linear DNA (Lin. DNA), plasmid DNA (PI. DNA) and genomic DNA (gDNA). PdeM had no effect on single stranded DNA (ss DNA) and RNA. Different treatments are shown above each lane.
(TIF)

S5 Fig Effect of metals on the oligomeric status of PdeM protein. Oligomeric status of the PdeM was determined with ~2 mg mL^-1^ of the purified protein which was resolved on Superdex 200 10/300 GL gel permeation column. 1.0 mL fractions were collected at a flow rate of 0.5 mL min^-1^. Protein was extracted from *E coli* BL21 (DE3) grown in the absence or presence of 100 μM Mn^2+^, Fe^3+^ or both.
(TIF)

S6 Fig Ramachandran Plot. (TIF)

S7 Fig Superimposition of homology model of PdeM (blue sticks) and Rv0805 (green sticks; PDBID: 3IB7_A), depicting the residues involved in dimer formation. Residues of PdeM occupy position at the outer surface. In PdeM, a bulky W243 is protruded out, which in turn may hamper the dimer formation. In order to accommodate a large loop connecting the β9 and β10, the β9 loop of PdeM retracts and adapts a conformation which is very much different from β9 of Rv0805.
(TIF)
