# Supplementary material for: Identification and Characterization of a Novel Phosphodiesterase from the Metagenome of an Indian Coalbed
Source: PLoS One. 2015 Feb 6;10(2):e0118075. doi: 10.1371/journal.pone.0118075 (PMC4320098; doi:10.1371/journal.pone.0118075)
Supplement: S1 Table — (DOC) [file pone.0118075.s001.doc]

**Table S1**

| **S. No.** | **Class I** | **Class II** | **Class III** | | |
| --- | --- | --- | --- | --- | --- |
|  | | | **Subclass IIIA** | **Subclass IIIB** | **Subclass IIIC** |
|  | gi|410968920 | gi|6831520 | gi|197295117 | gi|398350047 | **gi|52695568_YfcE** |
|  | gi|403258673 | gi|59711863 | gi|170734930 | gi|227820757 | gi|251785660 |
|  | gi|344268341 | gi|197334722 | gi|387904575 | gi|407719383 | gi|170680770 |
|  | gi|15128482 | gi|209695108 | gi|269128492 | gi|384534627 | gi|117624490 |
|  | gi|397489086 | gi|386327918 | gi|378950829 | gi|15964293 | gi|222157051 |
|  | gi|332209440 | gi|157369496 | gi|330809700 | gi|384528259 | gi|410481704 |
|  | gi|329664108 | gi|448240987 | gi|447917119 | gi|150395379 | gi|82777708 |
|  | gi|296204426 | gi|440229886 | gi|229591016 | gi|440225473 | gi|384543955 |
|  | gi|338715599 | gi|386309316 | gi|28867720 | gi|222084810 | gi|30063726 |
|  | gi|348585589 | gi|332160905 | gi|71734668 | gi|86356203 | gi|218548245 |
|  | gi|355565006 | gi|123443171 | gi|300717918 | gi|190890248 | gi|16761272 |
|  | gi|18266694 | gi|294504487 | gi|308187731 | gi|209547812 | gi|378954306 |
|  | gi|187957022 | gi|218929769 | gi|326318125 | gi|116250354 | gi|161502508 |
|  | gi|126326672 | gi|162418847 | gi|120612111 | gi|241202987 | gi|340000082 |
|  | gi|327284175 | gi|153950582 | gi|319761882 | gi|325291886 | gi|157144759 |
|  | gi|301610210 | gi|322833857 | gi|319792049 | gi|15887842 | gi|392980066 |
|  | gi|432935223 | gi|383191020 | gi|340788951 | gi|222147486 | gi|296103986 |
|  | gi|348519801 | gi|269119019 | gi|300309937 | gi|163844945 | gi|345300173 |
|  | gi|110815910 | gi|269139941 | gi|115351167 | gi|189022595 | gi|401764571 |
|  | gi|326670446 | gi|238920765 | gi|172060167 | gi|225686588 | gi|365971467 |
|  | gi|405958962 | gi|383317861 | gi|402567022 | gi|256015589 | gi|479273959 |
|  | gi|242014746 | gi|332304810 | gi|134295249 | gi|148557850 | gi|146312491 |
|  | gi|340374579 | gi|109896668 | gi|387901875 | gi|153011360 | gi|283786374 |
|  | gi|170061357 | gi|333892573 | gi|161525248 | gi|337271023 | gi|440286805 |
|  | gi|157106482 | gi|476411877 | gi|170732554 | gi|433777185 | gi|311278738 |
|  | gi|21483598 | gi|375145806 | gi|107022305 | gi|13474671 | gi|444350318 |
|  | gi|193904525 | gi|256423347 | gi|206559498 | gi|319785597 | gi|336251359 |
|  | gi|198474190 | gi|146298984 | gi|78065810 | gi|110632390 | gi|288934243 |
|  | gi|194149266 | gi|397667676 | gi|488601895 | gi|328544852 | gi|206576642 |
|  | gi|195437670 | gi|54297871 | gi|323526673 | gi|374330090 | gi|530629327 |
|  | gi|380017646 | gi|397664417 | gi|407714120 | gi|488575461 | gi|550445567 |
|  | gi|215510347 | gi|148359508 | gi|91784336 | gi|300021947 | gi|152971228 |
|  | gi|443696663 | gi|509151251 | gi|241663054 | gi|338737658 | gi|378979916 |
|  | gi|156379682 | gi|54294838 | gi|300691483 | gi|158421739 | gi|238895819 |
|  | gi|426345345 | gi|289164018 | gi|192290065 | gi|154245994 | gi|402779679 |
|  | gi|117645718 | gi|212218941 | gi|316935347 | gi|298290090 | gi|397659187 |
|  | gi|444721911 | gi|212213065 | gi|90422780 | gi|27376260 | gi|481848024 |
|  | gi|350587843 | gi|347759750 | gi|146342206 | gi|384214893 | gi|387888560 |
|  | gi|426231214 | gi|414342020 | gi|459288775 | gi|383774893 | gi|156933107 |
|  | gi|354498856 | gi|295689476 | gi|148257114 | gi|92115872 | gi|260598720 |
|  | gi|395541806 | gi|108760258 | gi|158422410 | gi|75674385 | gi|333928356 |
|  | gi|326918972 | gi|338533887 | gi|389879342 | gi|316932346 | gi|518649866 |
|  | gi|449499612 | gi|442320086 | gi|209964753 | gi|192289304 | gi|157371556 |
|  | gi|54038670 | gi|383457300 | gi|392378876 | gi|86751710 | gi|448243190 |
|  | gi|459181386 | gi|310820714 | gi|297561103 | gi|91975278 | gi|440231739 |
|  | gi|355749023 | gi|162451055 | gi|384147319 | gi|115522757 | gi|261820780 |
|  | gi|402868708 | gi|222055777 | gi|256377586 | gi|90422266 | gi|50121966 |
|  | gi|297679593 | gi|148263954 | gi|407641949 | gi|337742005 | gi|403059292 |
|  | gi|301787523 | gi|197117966 | gi|379706960 | gi|459287161 | gi|253689159 |
|  | gi|351704579 | gi|253701241 | gi|284031495 | gi|148252386 | gi|307131829 |
|  | gi|431904586 | gi|322418929 | **gi|15607945_Rv0805** | gi|146343502 | gi|271501279 |
|  | gi|432114979 | gi|116751502 | gi|433625888 | gi|218531411 | gi|251789002 |
|  | gi|449278039 | gi|206890602 | gi|340625820 | gi|163852571 | gi|242239980 |
|  | gi|47228972 | gi|85858176 | gi|433640922 | gi|254562329 | gi|45442173 |
|  | gi|376372938 | gi|374288586 | gi|433629887 | gi|188582592 | gi|384123100 |
|  | gi|308505622 | gi|347756771 | gi|494699194 | gi|170746972 | gi|294504635 |
|  | gi|158591046 | gi|300309651 | gi|183984854 | gi|170742378 | gi|218929852 |
|  | gi|333973287 | gi|91776480 | gi|118616266 | gi|220924624 | gi|384139377 |
|  | gi|328769586 | gi|372488124 | gi|221230664 | gi|114569082 | gi|332162404 |
|  | gi|402589371 | gi|257092295 | gi|296395375 | gi|182677370 | gi|386307638 |
|  | gi|312072524 | gi|71908970 | gi|54026843 | gi|374292339 | gi|285005769 |
|  | gi|3868997 | gi|302879753 | gi|325962027 | gi|288958780 | gi|387868300 |
|  | gi|17223618 | gi|339482267 | gi|220911475 | gi|392382532 | gi|269139746 |
|  | gi|261331205 | gi|325982780 | gi|116669127 | gi|154250910 | gi|470463444 |
|  | gi|12003123 | gi|30250218 | gi|119962173 | gi|295688805 | gi|238920595 |
|  | gi|71744548 | gi|114332094 | gi|226360768 | gi|221235059 | gi|320157077 |
|  | gi|342183290 | gi|313200387 | gi|323359390 | gi|167645418 | gi|27363676 |
|  | gi|71666626 | gi|253998313 | gi|226363107 | gi|197106223 | gi|37679179 |
|  | gi|37543960 | gi|253995981 | gi|119714899 | gi|304320139 | gi|218710240 |
|  | gi|70779485 | gi|56477796 | gi|323356800 | gi|357383446 | gi|28897590 |
|  | gi|407405394 | gi|470172052 | gi|317126730 | gi|148260612 | gi|433657076 |
|  | gi|56718344 | gi|74317002 | gi|111222424 | gi|326403806 | gi|262394913 |
|  | gi|340056027 | gi|220934023 | gi|86741523 | gi|114327565 | gi|156973619 |
|  | gi|146082226 | gi|289208145 | gi|312198502 | gi|334345851 | gi|375264771 |
|  | gi|322497894 | gi|124265259 | gi|407644499 | gi|294010001 | gi|147674558 |
|  | gi|40646535 | gi|171056881 | gi|378719289 | gi|148556450 | gi|379740810 |
|  | gi|389592920 | gi|383760494 | gi|433605543 | gi|87200550 | gi|384424149 |
|  | gi|401418359 | gi|121997107 | gi|152964042 | gi|103488504 | gi|375131652 |
|  | gi|154334650 | gi|78484479 | gi|257068050 | gi|347530156 | gi|336124684 |
|  |  | gi|73748267 | gi|251800020 | gi|221640115 | gi|170725325 |
|  |  | gi|289432316 | gi|251795905 | gi|77464230 | gi|157375864 |
|  |  | gi|270307793 | **gi|15803579_CpdA** | gi|146276625 | gi|54309962 |
|  |  |  | gi|170018714 | gi|159043667 | gi|59711396 |
|  |  |  | gi|386594243 | gi|89053476 | gi|197335053 |
|  |  |  | gi|384544632 | gi|56697931 | gi|209694389 |
|  |  |  | gi|260857165 | gi|339502795 | gi|157963870 |
|  |  |  | gi|386615818 | gi|110679385 | gi|167622140 |
|  |  |  | gi|170683396 | gi|478179567 | gi|308049336 |
|  |  |  | gi|218691335 | gi|400753454 | gi|153930976 |
|  |  |  | gi|215488365 | gi|399991812 | gi|226948272 |
|  |  |  | gi|386620656 | gi|119383168 | gi|153940984 |
|  |  |  | gi|218550281 | gi|407685441 | gi|387817280 |
|  |  |  | gi|205354099 | gi|407689380 | gi|170757313 |
|  |  |  | gi|488655567 | gi|407701667 | gi|170759843 |
|  |  |  | gi|56415124 | gi|410863333 | gi|28211458 |
|  |  |  | gi|161616172 | gi|333895368 | gi|488770804 |
|  |  |  | gi|340000705 | gi|348030479 | gi|407474854 |
|  |  |  | gi|161506251 | gi|315498379 | gi|15896006 |
|  |  |  | gi|157148597 | gi|312113324 | gi|117620056 |
|  |  |  | gi|283787163 | gi|414344099 | gi|330830312 |
|  |  |  | gi|336247218 | gi|255037086 | |
|  |  |  | gi|444354668 | gi|387790304 | |
|  |  |  | gi|378980732 | gi|110639892 | |
|  |  |  | gi|206579155 | gi|375013250 | |
|  |  |  | gi|375257403 | gi|256425814 | |
|  |  |  | gi|397660012 | gi|298207039 | |
|  |  |  | gi|481852209 | gi|332291671 | |
|  |  |  | gi|311278048 | gi|120437477 | |
|  |  |  | gi|440286127 | gi|295132115 | |
|  |  |  | gi|296104692 | gi|390953907 | |
|  |  |  | gi|392980691 | gi|319953410 | |
|  |  |  | gi|401765235 | gi|344202967 | |
|  |  |  | gi|365972111 | gi|146299517 | |
|  |  |  | gi|345300850 | gi|408491152 | |
|  |  |  | gi|146313081 | gi|443242840 | |
|  |  |  | gi|449306918 | gi|260060949 | |
|  |  |  | gi|260599302 | gi|313674887 | |
|  |  |  | gi|387887893 | gi|326798531 | |
|  |  |  | gi|403056769 | gi|472322516 | |
|  |  |  | gi|253686722 | gi|336173729 | |
|  |  |  | gi|50119292 | gi|255532458 | |
|  |  |  | gi|261819680 | gi|325104202 | |
|  |  |  | gi|470152837 | gi|312129498 | |
|  |  |  | gi|307129108 | gi|239817816 | |
|  |  |  | gi|251791332 | gi|319796121 | |
|  |  |  | gi|271498903 | **gi|528880715_PdeM** | |
|  |  |  | gi|242241015 | gi|120609080 | |
|  |  |  | gi|386309930 | gi|326315265 | |
|  |  |  | gi|332162980 | gi|160898487 | |
|  |  |  | gi|123443847 | gi|333915287 | |
|  |  |  | gi|294502733 | gi|222109528 | |
|  |  |  | gi|384125046 | gi|121592747 | |
|  |  |  | gi|108810689 | gi|300311591 | |
|  |  |  | gi|162421040 | gi|124266709 | |
|  |  |  | gi|22127387 | gi|407936841 | |
|  |  |  | gi|153949136 | gi|330503432 | |
|  |  |  | gi|51597695 | gi|146307220 | |
|  |  |  | gi|170022908 | gi|386021382 | |
|  |  |  | gi|384416267 | gi|339494723 | |
|  |  |  | gi|157372498 | gi|392420869 | |
|  |  |  | gi|333929204 | gi|431926792 | |
|  |  |  | gi|448243951 | gi|397687592 | |
|  |  |  | gi|440232579 | gi|229588837 | |
|  |  |  | gi|322831361 | gi|447915671 | |
|  |  |  | gi|383188592 | gi|387892489 | |
|  |  |  | gi|269137551 | gi|77457431 | |
|  |  |  | gi|238918164 | gi|330807869 | |
|  |  |  | gi|85058247 | gi|66047108 | |
|  |  |  | gi|386077939 | gi|71737903 | |
|  |  |  | gi|291618923 | gi|28871284 | |
|  |  |  | gi|308188125 | gi|333900269 | |
|  |  |  | gi|317049583 | gi|395447568 | |
|  |  |  | gi|292900682 | gi|386010762 | |
|  |  |  | gi|387869840 | gi|148546385 | |
|  |  |  | gi|188532589 | gi|397694602 | |
|  |  |  | gi|300718431 | gi|26987838 | |
|  |  |  | gi|253991003 | gi|167035303 | |
|  |  |  | gi|37527807 | gi|339489044 | |
|  |  |  | gi|290474838 | gi|431804093 | |
|  |  |  | gi|300724861 | gi|170720317 | |
|  |  |  | gi|386744262 | gi|194292338 | |
|  |  |  | gi|197286193 | gi|339323148 | |
|  |  |  | gi|455738030 | gi|73541541 | |
|  |  |  | gi|148825320 | gi|119896737 | |
|  |  |  | gi|378696582 | gi|383756888 | |
|  |  |  | gi|68249002 | gi|337278526 | |
|  |  |  | gi|16272348 | gi|226228807 | |
|  |  |  | gi|386265206 | gi|238024154 | |
|  |  |  | gi|386833812 | gi|330815885 | |
|  |  |  | gi|15602884 | gi|377819963 | |
|  |  |  | gi|383309830 | gi|407709346 | |
|  |  |  | gi|262395288 | gi|307727226 | |
|  |  |  | gi|28897202 | gi|187919174 | |
|  |  |  | gi|156973192 | gi|91779319 | |
|  |  |  | gi|37678770 | gi|295700064 | |
|  |  |  | gi|360036307 | gi|186471501 | |
|  |  |  | gi|147675382 | gi|156741324 | |
|  |  |  | **gi|24210990_PdeA** | gi|148657652 | |
|  |  |  |  | gi|108761694 | |
|  |  |  |  | gi|338532825 | |
|  |  |  |  | gi|383458568 | |
|  |  |  |  | gi|310823995 | |
|  |  |  |  | gi|383766242 | |
|  |  |  |  | gi|58581496 | |
|  |  |  |  | gi|188577265 | |
|  |  |  |  | gi|384420071 | |
|  |  |  |  | gi|84623426 | |
|  |  |  |  | gi|346724235 | |
|  |  |  |  | gi|78046948 | |
|  |  |  |  | gi|21242092 | |
|  |  |  |  | gi|384427202 | |
|  |  |  |  | gi|21230745 | |
|  |  |  |  | gi|188992410 | |
|  |  |  |  | gi|319785881 | |
|  |  |  |  | gi|190575398 | |
|  |  |  |  | gi|344208395 | |
|  |  |  |  | gi|386719477 | |
|  |  |  |  | gi|194366734 | |
|  |  |  |  | gi|296120627 | |
|  |  |  |  | gi|325110164 | |
|  |  |  |  | gi|32476508 | |
|  |  |  |  | gi|148239507 | |
|  |  |  |  | gi|427702071 | |
|  |  |  |  | gi|148242114 | |
|  |  |  |  | gi|78779115 | |
|  |  |  |  | gi|123968316 | |
|  |  |  |  | gi|157413150 | |
|  |  |  |  | gi|123966094 | |
