# Supplementary figures and images for: Identification and Characterization of a Novel Phosphodiesterase from the Metagenome of an Indian Coalbed
Source: PLoS One. 2015 Feb 6;10(2):e0118075. doi: 10.1371/journal.pone.0118075 (PMC4320098; doi:10.1371/journal.pone.0118075)

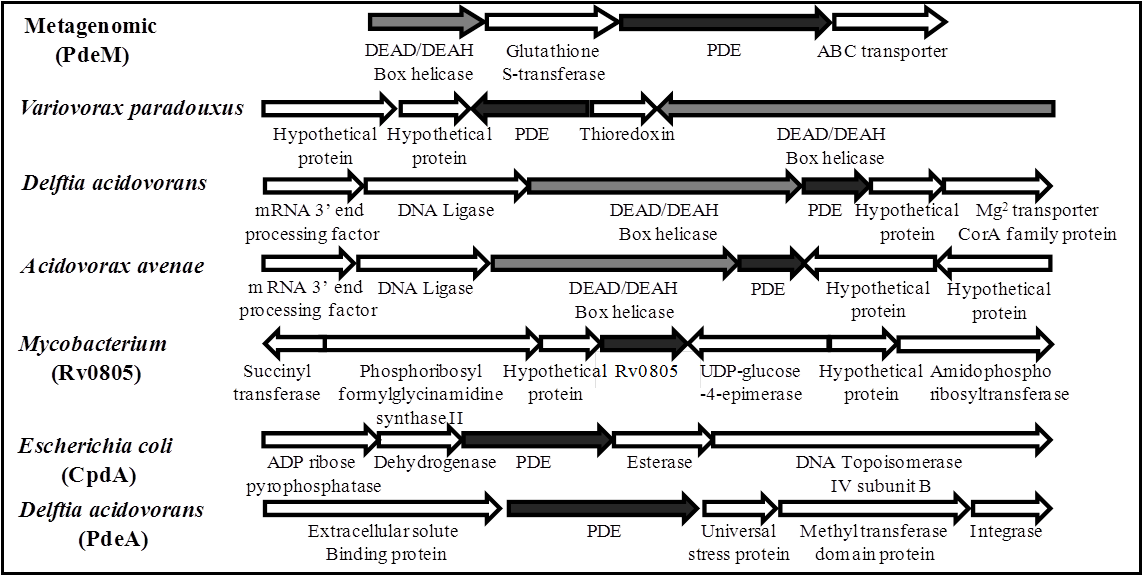

Supplement: S1 Fig — (TIF) [file pone.0118075.s002.tif]

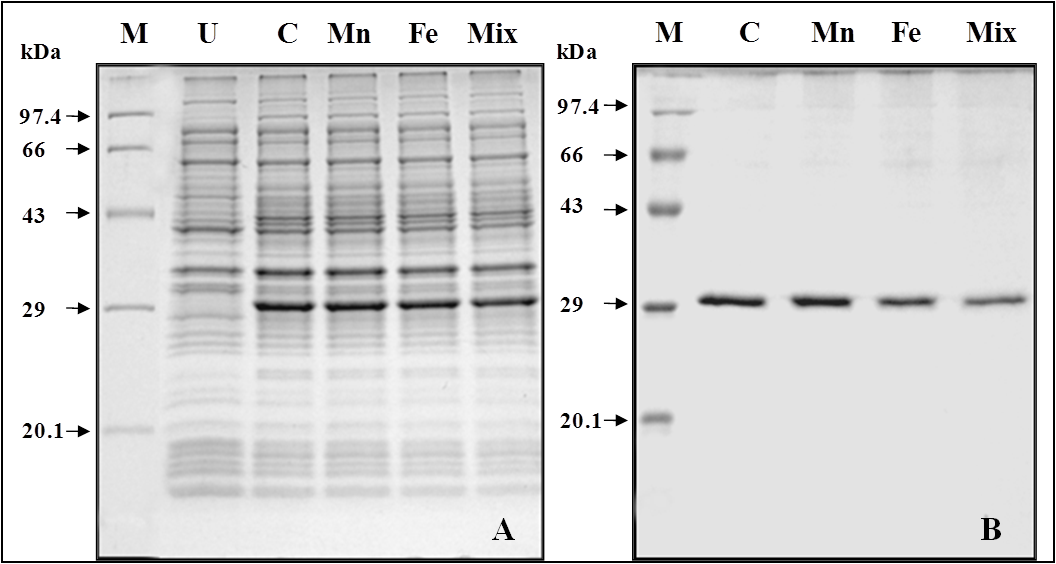

Supplement: S2 Fig — (A) 12% SDS-PAGE depicting overexpression. (B) Ni-NTA affinity-purification of PdeM extracted from the E. coli BL21 (DE3) grown in the absence or presence of 100 μM Mn2+, Fe3+ or both. U- Uninduced cells of E. coli BL21 (DE3); C- E. coli BL21 (DE3) harbouring the pET-PdeM induced with 0.5 mM IPTG at 16°C for 20 hour; Mn- PdeM overexpressed in presence of 100 μM Mn2+; Fe- PdeM overexpressed in presence of 100 μM Fe3+; Mix- PdeM overexpressed in presence of 100 μM of Mn2+ and Fe3+ each, and M- Protein molecular size marker (14–97.4 kDa). (TIF) [file pone.0118075.s003.tif]

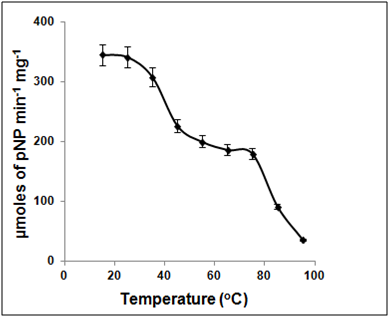

Supplement: S3 Fig — (TIF) [file pone.0118075.s004.tif]

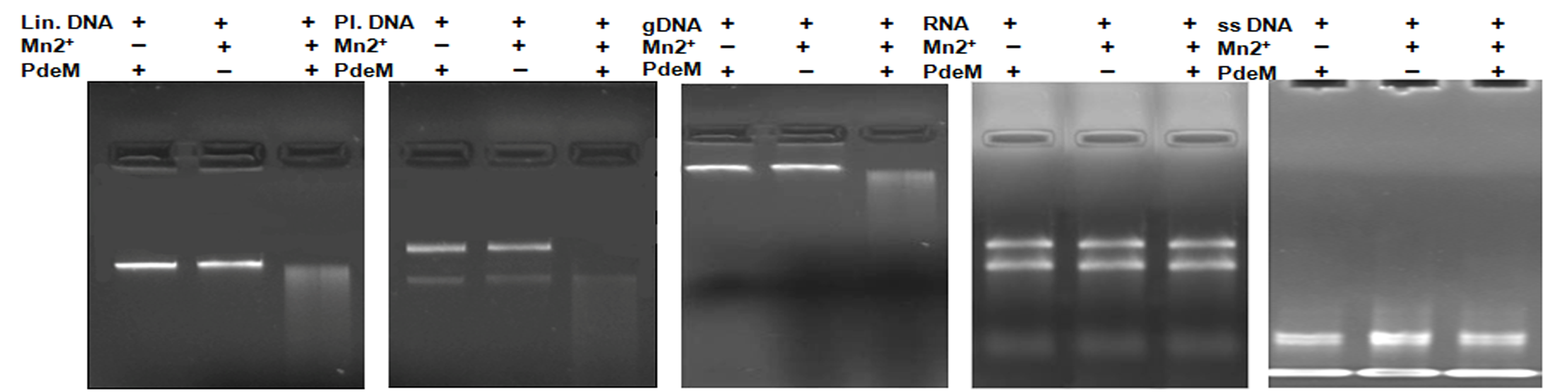

Supplement: S4 Fig — PdeM had no effect on single stranded DNA (ss DNA) and RNA. Different treatments are shown above each lane. (TIF) [file pone.0118075.s005.tif]

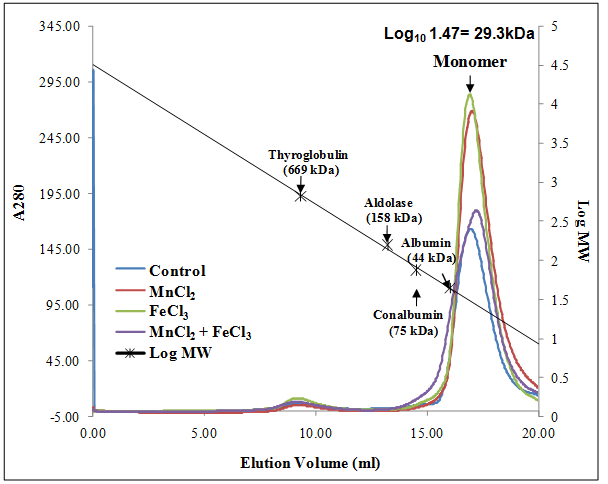

Supplement: S5 Fig — Oligomeric status of the PdeM was determined with ~2 mg mL-1 of the purified protein which was resolved on Superdex 200 10/300 GL gel permeation column. 1.0 mL fractions were collected at a flow rate of 0.5 mL min-1. Protein was extracted from E coli BL21 (DE3) grown in the absence or presence of 100 μM Mn2+, Fe3+ or both. (TIF) [file pone.0118075.s006.tif]

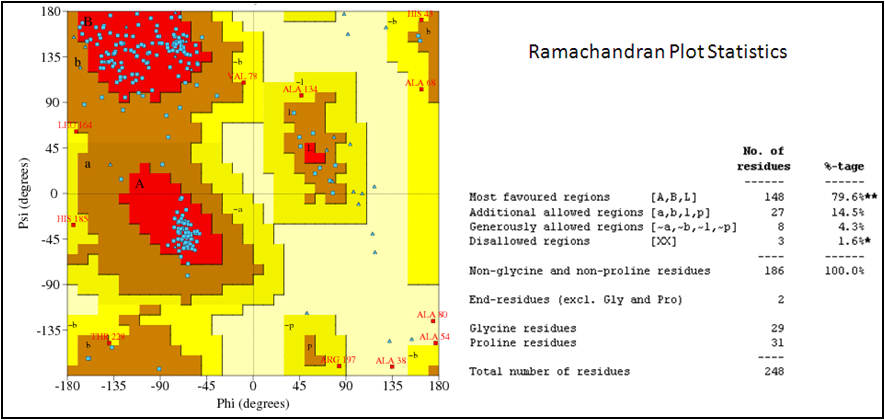

Supplement: S6 Fig — (TIF) [file pone.0118075.s007.tif]

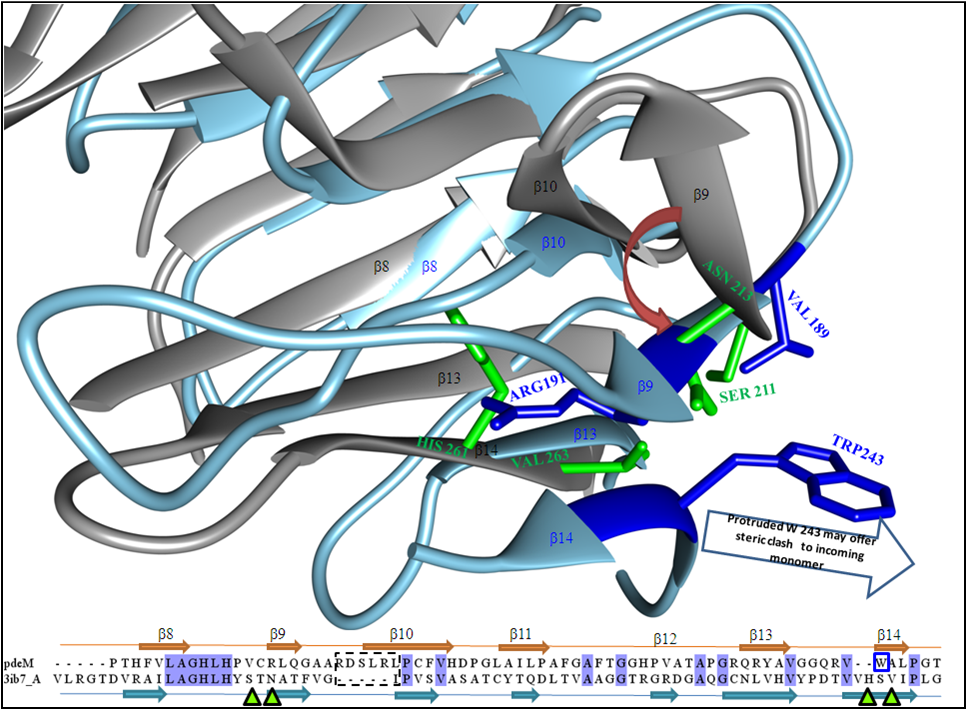

Supplement: S7 Fig — Residues of PdeM occupy position at the outer surface. In PdeM, a bulky W243 is protruded out, which in turn may hamper the dimer formation. In order to accommodate a large loop connecting the β9 and β10, the β9 loop of PdeM retracts and adapts a conformation which is very much different from β9 of Rv0805. (TIF) [file pone.0118075.s008.tif]
